# Characterization of 4‑Bromo-2,5-dimethoxyphenethyl Tosylate, a Synthetic Intermediate of 2C–B, Identified in a Seized Pink Heart-Shaped Tablet

**DOI:** 10.1021/acs.chemrestox.6c00170

**Published:** 2026-08-24

**Authors:** Sara Casati, Roberta F. Bergamaschi, Michele Dei Cas, Alessandro Ravelli, Lea Sicuro, Gabriella Roda, Chiara Ciccarelli, Luca Mollica, Claudio Guidotti, Alessio Battistini, Paola Rota

**Affiliations:** † Dipartimento di Scienze Biomediche, Chirurgiche ed Odontoiatriche, 9304 Università degli Studi di Milano, 20133 Milan, Italy; ‡ Fondazione IRCCS Ca’ Granda Ospedale Maggiore Policlinico, 20122 Milan, Italy; § Dipartimento di Scienze della Salute, Università degli Studi di Milano, 20142 Milan, Italy; ∥ Dipartimento di Biotecnologie Mediche e Medicina Traslazionale, Università degli Studi di Milano, 20054 Segrate, Italy; ⊥ Dipartimento di Scienze Farmaceutiche, Università degli Studi di Milano, 20133 Milan, Italy; # Gabinetto Regionale di Polizia Scientifica per la Lombardia, Polizia di Stato, Via Fatebenefratelli 11, 20121 Milan, Italy; ∇ Institute for Molecular and Translational Cardiology (IMTC), San Donato Milanese, 20097 Milan, Italy

## Abstract

A pink heart-shaped tablet, seized by law enforcement authorities, was found to contain a compound structurally related to 4-bromo-2,5-dimethoxyphenethylamine (2C–B), a new psychoactive substance (NPS). An extensive analytical investigation based on one- and two-dimensional nuclear magnetic resonance (NMR) experiments, combined with liquid chromatography coupled to high-resolution mass spectrometry (LC–HRMS) and gas chromatography coupled to mass spectrometry (GC–MS) analyses, enabled the unambiguous identification of a new compound as 4-bromo-2,5-dimethoxyphenethyl tosylate, a putative synthetic intermediate in the synthesis of 2C–B. Finally, the targeted synthesis of the proposed structure provided independent confirmation of the correct structural assignment.

## Graphical abstract

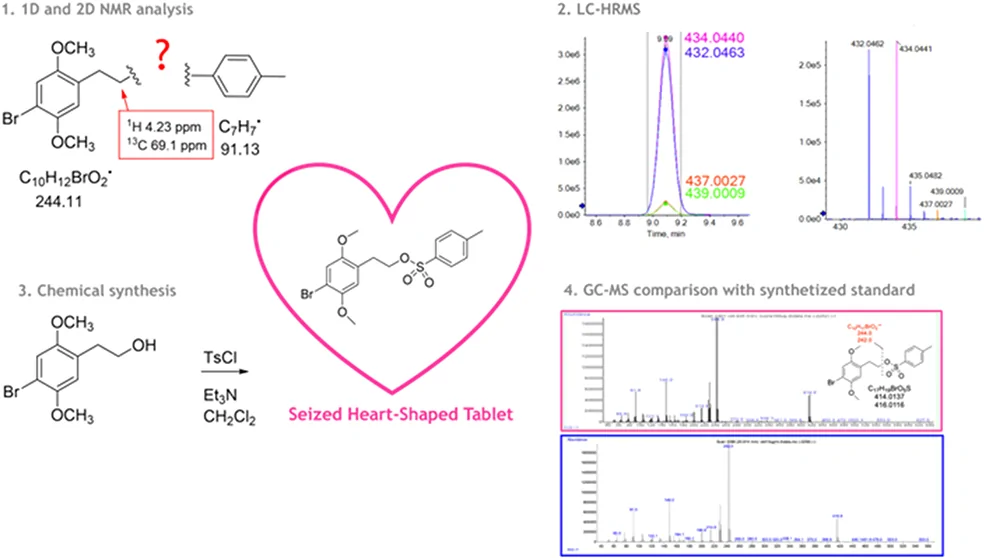

## 1 Introduction

The phenomenon of new psychoactive substances (NPSs) represents a major and evolving challenge for public health, forensic laboratories, and regulatory authorities worldwide.^1,2^ NPSs comprise a broad and heterogeneous group of compounds designed to mimic the pharmacological effects of controlled drugs while circumventing existing legal frameworks.^2,3^ Owing to their recent emergence and structural diversity, the toxicological profiles of many NPS remain poorly characterized, with several substances being associated with severe or fatal intoxications.^3^ Within the class of phenethylamines, 4-bromo-2,5-dimethoxyphenethylamine (2C–B, Figure 1) is a well-known psychoactive compound belonging to the 2C-series, which shares a 2,5-dimethoxyphenethylamine scaffold bearing a lipophilic substituent at the 4-position that critically modulates potency and receptor selectivity.^4^ 2C–B exhibits mixed serotonergic activity, acting primarily as an agonist at 5-HT_2A_ and 5-HT_2C_ receptors, and produces combined hallucinogenic and stimulant effects.^5–8^ Because of its abuse potential and associated health risks, 2C–B is subject to international and national control measures. However, as observed for other controlled substances, NPS legislative scheduling has driven the appearance of structurally related derivatives, precursors, and synthetic intermediates that are not explicitly listed in current regulations and can therefore transiently evade legal control.^3,9^

**Figure 1:**
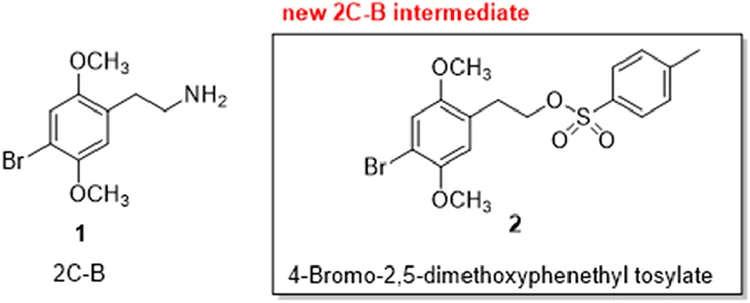
Chemical structures of 2C–B and 2C–B phenethyl tosylate intermediate.

Indeed, the illicit manufacture of NPS relies on precursors, masked intermediates, and versatile starting materials that can be rapidly adapted to generate multiple end products within a structural class, including phenethylamines.^3^ Consequently, policy responses increasingly combine substance-specific scheduling with broader framework measures (such as generic definitions, analogue provisions, and extended precursor monitoring systems). However, available data on the actual market diffusion of individual NPS precursors remain largely qualitative, consisting mainly of case reports, early warning notifications, and targeted operations rather than comprehensive prevalence data.^2^

From an analytical perspective, the continuous structural modification of NPSs poses significant challenges for their identification, particularly when reference standards are unavailable and mass spectral libraries are incomplete or outdated.^10–15^ Routine analytical approaches for NPS identification rely primarily on gas chromatography (GC) or liquid chromatography (LC) coupled to low- and high-resolution (HR) mass spectrometry (MS). Nevertheless, when newly emerging substances are encountered, MS data alone may be insufficient to achieve an unambiguous structural assignment. In such cases, the combined use of HRMS and one- and two-dimensional nuclear magnetic resonance (NMR) spectroscopy is often required to obtain complementary information on elemental composition and molecular connectivity.^10–15^

In the present study, a pink heart-shaped tablet seized by law enforcement authorities was found to contain a compound structurally related to 2C–B. An extensive analytical investigation was undertaken, combining GC–MS analyses, liquid chromatography coupled to high-resolution mass spectrometry (LC-HRMS), and comprehensive one-dimensional nuclear magnetic resonance (1D NMR) and two-dimensional nuclear magnetic resonance (2D NMR) experiments. This approach enabled the identification of the compound as 4-bromo-2,5-dimethoxyphenethyl tosylate (Figure 1), interpreted as a putative synthetic intermediate in the production of 2C–B derivatives. The proposed structure was further corroborated by targeted chemical synthesis, providing orthogonal confirmation of the molecular assignment.

## 2 Results and Discussion

Initial gas chromatography coupled to mass spectrometry (GC-MS) analysis of the seized tablet revealed a brominated phenethyl-based structure whose fragmentation pattern could not be unequivocally assigned to any known substance in available spectral libraries (see Supporting Information). To achieve structural elucidation, the sample was therefore subjected to a comprehensive analytical investigation, combining NMR, LC-HRMS, and GC-MS analyses.

Comparison of the ^1^H NMR spectra recorded in CDCl_3_ and CD_3_OD (see the Supporting Information) allowed the discrimination between signals attributable to tablet excipients and those associated with the target compound having differential solubility behavior. Specifically, the signals of excipients were prominent in CD_3_OD, conversely, they were significantly reduced in CDCl_3_, consistent with excipients soluble in methanol but poorly soluble in chloroform.

To further assist in the interpretation of excipient-related signals observed in CD_3_OD, a ^1^H NMR spectrum of 2C–B obtained from a pink tablet formulation seized in a separate case was acquired for comparison. The excipient-related signals were found to be analogous in both formulations, enabling the exclusion of several high-field resonances attributable to common tablet excipients, such as fatty acid esters (e.g., stearates or palmitates). The presence of these excipients was further corroborated by GC–MS analysis of the seized material (see the Supporting Information).

Analysis of the ^1^H, ^13^C, correlation spectroscopy (COSY), heteronuclear single quantum coherence (HSQC), and heteronuclear multiple bond correlation (HMBC) spectra led to the unambiguous identification of two structural fragments, hereafter termed fragments A and B (Figure 2). Integration of the NMR signals associated with fragments A and B shows that the two fragments are present in a constant 1:1 ratio in both solvents (CD_3_OD and CDCl_3_). This observation suggests that fragments A and B either belong to the same molecule or, less likely, to two distinct molecules present in equimolar amounts and exhibiting very similar solubilities in the two solvents used.

**Figure 2:**
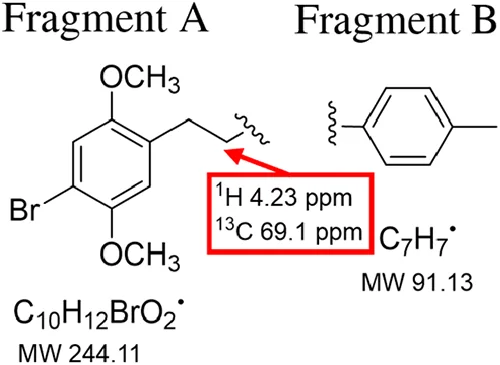
Structural fragments A and B identified by combined 1D and 2D NMR analyses, exhibiting a constant 1:1 ratio by integration in both CD_3_OD and CDCl_3_.

Fragment A shows NMR features consistent with a dimethoxy-substituted phenethyl moiety. Two isolated aromatic singlets at δ 6.89 and 6.64 indicate a highly substituted aromatic ring. Two methoxy groups are identified by singlets at δ 3.80 and 3.65 ppm, supported by the corresponding ^13^C resonances at δ 56.9 and 55.9 ppm. The aliphatic region displays two coupled triplets at δ 4.23 and 2.89 ppm, consistent with a −CH_2_–CH_2_– fragment, with the downfield methylene (δ_C_ 69.1 ppm) indicative of oxygen substitution.

Fragment B exhibits a ^1^H NMR spectral pattern characteristic of a para-disubstituted benzene ring, showing an AA′BB′ spin system. Two aromatic doublets integrating for two protons each are observed at δ 7.60 (2H, d, *J* = 8.3 Hz) and δ 7.24 (2H, d, *J* = 8.3 Hz). A singlet at δ 2.44 (3H, s, CH_3_) is assigned to an aromatic methyl group, consistent with a *p*-tolyl moiety, as supported by the corresponding ^13^C resonance at δ 21.7 ppm. Aromatic CH carbons appear at δ 129.6 and 127.8, while the more deshielded signals at δ 144.5 and 132.8 are attributed to quaternary aromatic carbons, including the carbon bonded to an electron-withdrawing substituent. Complete ^1^H and ^13^C NMR spectra, together with full signal assignments and two-dimensional NMR data, are provided in Section 3 and in the Supporting Information sections.

After the structural elucidation of fragments A and B by NMR spectroscopy, the key unresolved issue was their mutual connectivity.

GC–MS analysis under electron ionization (EI) conditions showed molecular ion peaks at *m*/*z* 414 and 416, displaying the characteristic isotopic pattern of a monobrominated compound and indicating a nominal molecular weight of 414/416 u. The molecular weights estimated for fragments A (244.11 u) and B (91.13 u) from NMR data account for a combined mass of 335.24 u. This value is approximately 79 u lower than the molecular ion observed by GC–MS, strongly suggesting the presence of an additional structural moiety linking the two fragments within a single molecular entity.

To define the elemental composition of the intact molecule and identify the missing structural portion suggested by GC–MS analysis, LC–HRMS was performed. Two pairs of precursor ions exhibiting the characteristic bromine isotopic pattern were detected at *m*/*z* 432.0462/434.0441 and 437.0027/439.0009, coeluting at identical retention time (see Figure 3, Panels A, C, E, G). The first ion pair was approximately 40 times more intense than the second. Despite their different nominal masses, all precursor ions generated identical MS/MS spectra (Figure 3, Panels B, D, F, H), demonstrating that they originated from the same neutral molecular species.

**Figure 3:**
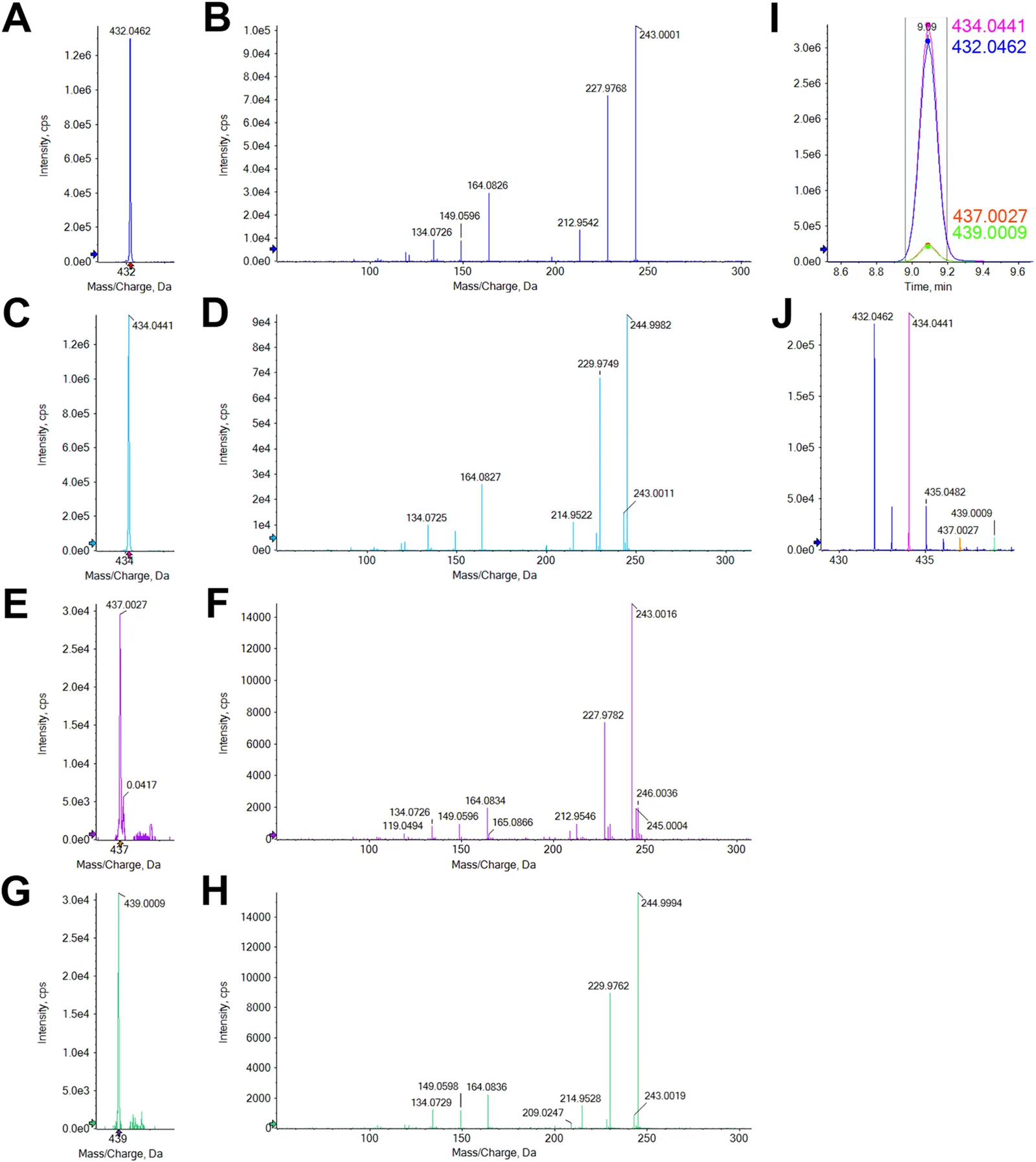
Untargeted LC–HRMS analysis of the unknown tablet. The chromatographic peak at 9.1 min (I) shows two pairs of precursor ions at *m*/*z* 434.0441 (^81^Br) and 432.0462 (^79^Br) (A, C), assigned to the [M + NH_4_]^+^ adduct, and at *m*/*z* 439.0009 (^81^Br) and 437.0027 (^79^Br) (E, G), assigned to the [M + Na]^+^ adduct. The corresponding MS/MS spectra (B, D, F, H) display the same fragmentation pattern. Full-scan mass spectrum of the chromatographic peak at 9.1 min, showing both the [M + NH_4_]^+^ and [M + Na]^+^ adduct ion pairs (J).

Accurate mass interpretation was carried out using an in-house Visual Basic for Excel program, applying a mass tolerance of ±0.0015 u (corresponding to 3.4 ppm at *m*/*z* 440). For the isotopic pair at *m*/*z* 437.0027/439.0009, a single elemental composition was obtained, corresponding to C_17_H_19_O_5_SBrNa^+^ (calcd 437.0029 and 439.0008).

Similarly, for the ion pair at *m*/*z* 432.0462/434.0441, a unique elemental composition was assigned, corresponding to C_17_H_23_O_5_SNBr^+^ (calcd 432.0475 and 434.0454), which is fully consistent with the expected isotopic pattern of bromine.

Subtraction of the elemental composition attributable to fragments A and B (C_17_H_19_O_2_Br) from the calculated ionic formulas yielded H_4_NO_3_S^+^ for the 432/434 pair and NaO_3_S^+^ for the 437/439 pair. These results indicate the presence of an SO_3_ moiety associated with NH_4_^+^ and Na^+^, respectively, identifying the detected species as ammonium and sodium adducts.

Removal of the adduct components led to the neutral molecular formula C_17_H_19_O_5_SBr (MW 414/416 u), fully consistent with the molecular ion observed by GC–MS (EI) and confirming the presence of a sulfonyl linker bridging fragments A and B. The compound was thus identified as 4-bromo-2,5-dimethoxyphenethyl tosylate (2) (Figure 1), a plausible synthetic intermediate in the preparation of 2C–B derivatives.

All fragment ions observed in the LC–HRMS/MS experiments were consistent with the presence of fragment A (see the Supporting Information), whereas no diagnostic product ions clearly attributable to fragment B were detected. MS/MS fragmentation of all precursor ions consistently generated a common set of product ions, including bromine-containing fragments at *m*/*z* 245/243, 230/228, and 214/212, as well as bromine-free ions at *m*/*z* 164 and 134. The recurrence of identical major product ions across all precursor species further supports their origin from a single molecular structure. Finally, considering that the proposed compound is unlikely to represent a psychoactive substance per se, but rather a putative synthetic precursor, and noting that it has not been previously reported in major chemical databases (SciFinder and Reaxys), a targeted synthesis was undertaken. The compound was synthesized starting from the corresponding alcohol, 2-(4-bromo-2,5-dimethoxyphenyl) ethan-1-ol, which was reacted with *p*-toluenesulfonyl chloride in the presence of Et_3_N as an acid scavenger (Figure 4). The target molecule was obtained in good yield (67%) after chromatographic purification and was fully characterized.

**Figure 4:**
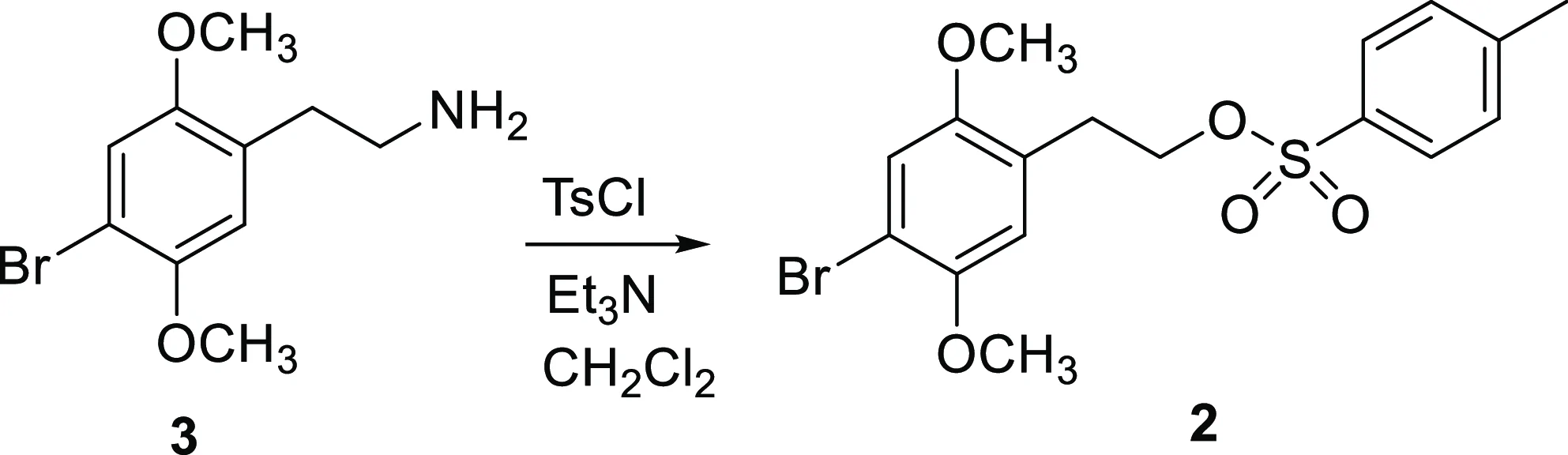
Synthesis of 4‑bromo-2,5-dimethoxyphenethyl tosylate: The alcohol, 2-(4-bromo-2,5-dimethoxyphenyl) ethan-1-ol, was reacted with *p*-toluenesulfonyl chloride (TsCl) in the presence of triethylamine (Et_3_N) overnight at room temperature.

In addition to identical retention times in GC and LC analyses, the NMR spectra and the HRMS and HRMS/MS profiles (see the Supporting Information) of the synthesized compound were fully superimposable with those obtained from the seized sample, providing definitive confirmation of the proposed structure.

Moreover, given the central role of GC–MS (EI) in forensic toxicology laboratories for the identification of emerging substances, the EI spectrum of compound **2** was compared with the reference spectrum of 2C–B available in the mass spectral library. The molecular ion at *m*/*z* 414/416 was clearly observed and represents a diagnostic feature of compound **2**, immediately distinguishing it from underivatized 2C–B. A highly intense fragment ion pair at *m*/*z* 242/244 (100% relative intensity) was detected for compound **2**; this ion is characteristic of *N*-derivatized 2C–B analogues (e.g., acetylated or perfluorinated propionate derivatives)^16^ but is absent or present at very low intensity (<5%) in the EI spectrum of nonderivatized 2C–B. Similarly, the ion pairs at *m*/*z* 229/231 and 227/229, together with the fragment at *m*/*z* 148 (30% relative intensity), were significantly more prominent in compound **2** and its *N*-derivatives than in 2C–B itself. In contrast, fragment ions at *m*/*z* 199/201, 65, 77, and 91 were common to both spectra, reflecting the shared brominated phenethyl core. Overall, the combined presence of the molecular ion at 414/416 and the diagnostic fragments at 242/244, 229/231, 227/229, and 148 provides a clear analytical basis for differentiating the tosylated intermediate from 2C–B in routine GC–MS workflows. Furthermore, the synthesized reference standard was also characterized by low-field (80 MHz) ^1^H and ^13^C NMR spectroscopy to facilitate routine identification in forensic laboratories, where low-resolution NMR instruments are commonly available to law enforcement agencies (see the Supporting Information).

## 3 Materials and Methods

### 3.1 Chemicals

All chemicals and solvents used were of analytical grade and purchased from Sigma-Aldrich (St. Louis, MO, USA).

### 3.2 NMR

#### 3.2.1 Sample Preparation for NMR Analysis

Half of the seized pink tablet was crushed and homogenized using a mortar and pestle to obtain a fine powder. An aliquot of the resulting powder was mixed directly with the appropriate deuterated solvent (CDCl_3_ or CD_3_OD), and the resulting suspension was transferred to an NMR tube. The sample was allowed to settle prior to analysis, and the supernatant was used for the NMR data acquisition.

##### 3.2.1.1 High-Field NMR Spectroscopy

Nuclear magnetic resonance spectra were recorded at 298 K on a Bruker AM-500 spectrometer equipped with a 5 mm inverse-geometry broadband probe and operating at 500.13 MHz for ^1^H and 125.76 MHz for ^13^C. Chemical shifts are reported in parts per million and are referenced for ^1^H spectra to a solvent residue proton signal (δ = 3.31 ppm for CD_3_OD or δ = 7.26 ppm for CDCl_3_) and for ^13^C spectra to solvent carbon signal (central line at 49.05 ppm, for CD_3_OD or δ = 77.0 ppm for CDCl_3_).

Proton and carbon assignments were established with ^1^H–^1^H and ^1^H–^13^C correlated NMR experiments. ^1^H NMR data are tabulated in the following order: number of protons, multiplicity (s, singlet; d, doublet; t, triplet, t app, apparent triplet, br s, broad singlet; m, multiplet), coupling constant(s) in hertz, and assignment of proton(s).

The numbering was done according to Figure 5

**Figure 5:**
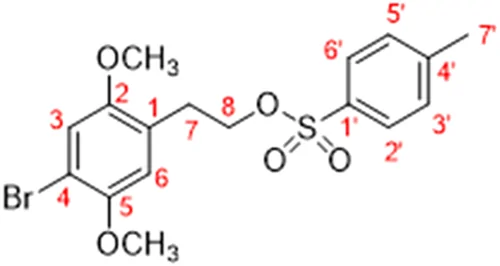
Chemical structure of 4-bromo-2,5-dimethoxyphenethyl tosylate (2), with atom numbering.

### 3.3 NMR of Compound **2**

Compound **2** ^1^H NMR (500 MHz, CDCl_3_): δ 7.60 (2H, d, *J* = 8.3 Hz, H-2′ and H-6′), 7.24 (2H, d, *J* = 8.3 Hz, H-3′ and H-5′), 6.89 (1H, s, H-3), 6.64 (1H, s, H-6), 4.23 (2H, t, *J* = 6.5 Hz, H-8a and H-8b), 3.80 (3H, s, OCH_3_ at C-5), 3.65 (3H, s, OCH_3_ at C-2), 2.89 (2H, t, *J* = 6.5 Hz, H-7a and H-7b), 2.44 (3H, s, CH_3_ at 7′); ^1^H NMR (500 MHz, CD_3_OD): δ 7.50 (2H, d, *J* = 8.3 Hz, H-2′ and H-6′), 7.28 (2H, d, *J* = 8.3 Hz, H-3′ and H-5′), 6.94 (1H, s, H-3), 6.71 (1H, s, H-6), 4.23 (2H, t, *J* = 6.5 Hz, H-8a and H-8b), 3.75 (3H, s, OCH_3_ at C-5), 3.64 (3H, s, OCH_3_ at C-2), 2.86 (2H, t, *J* = 6.5 Hz, H-7a and H-7b), 2.44 (3H, s, CH_3_ at 7′); ^13^C NMR (125.76 MHz, CDCl_3_): δ 151.7 (C2), 149.6 (C5), 144.5 (C4′), 132.8 (C1′), 129.6 (2C, C5′ and 3′), 127.8 (2C, C2′ and 6′), 124.7 (C1), 115.6 (C3), 115.4 (C6), 110.0 (C4), 69.1 (C8), 56.9 (OCH_3_ at C5), 55.9 (OCH_3_ at C2), 30.6 (C7), 21.7 (C7′).

#### 3.3.1 Low-Field NMR Spectroscopy

Nuclear magnetic resonance spectra of the synthesized standard compound **2** were recorded at 298 K on a Bruker Fourier 80 benchtop spectrometer operating at 80 MHz for ^1^H. Chemical shifts (δ) are reported in parts per million (ppm) and referenced to the residual solvent proton signal (δ = 7.26 ppm for CDCl_3_); ^13^C spectra were referenced to the solvent carbon signal (central line at δ = 77.0 ppm for CDCl_3_). One- and two-dimensional NMR experiments were performed, including ^1^H, ^13^C, COSY, and nuclear Overhauser effect spectroscopy (NOESY), to support structural assignment (see the Supporting Information).

### 3.4 LC/HRMS

LC-HRMS and MS/MS analyses were performed using a Shimadzu LC40 XR UHPLC system coupled with a Sciex Triple TOF X500B (Concord, ON, CA) equipped with a Turbo V ion source. The sample was analyzed in positive electrospray ionization (ESI) mode. Spectra were simultaneously acquired by a full-scan TOF-MS from 50 to 600 *m*/*z* and a top-25 data-dependent acquisition (DDA) with dynamic background subtraction from 50 to 600 *m*/*z*. The accumulation times were 100 mf for for TOF-MS and 40 ms for TOF MS/MS 40, resulting in a total scan time of less than 1 s. Declustering potential was fixed to 40 V, and the collision energy was 35 ± 10 eV. The electrospray ionization voltage was set at 5.5 kV. The source and gas parameters were set as follows: gas 1 55 psi, gas 2 65 psi, curtain gas 35 psi, and temperature 500 °C. The chromatographic separation was reached on a reversed-phase column C18 Kinetex 2.6 μm, 100x 2.1 mm (Phenomenex, Torrance, CA, USA) equipped with a precolumn. The column temperature was 55 °C and the flow rate was 0.3 mL/min. The mobile phases consisted of (A) water and (B) acetonitrile, both cotaining 0.1% formic acid. Gradient details were the following (%B): 0–1.5 min 5%, 1.5–2.0 min 5–20%, 2.0–4.5 min 20.0–35.0%, 4.5–6.0 min 35%, 6.0–9.0 min 35–99%, and 9.0–12.0 min 99%, then re-equilibrated at 5% until 15 min.

### 3.5 GC/MS

The GC separation was carried out on an Agilent capillary column Varian CP-Sil8 (15 m length ×  0.25 mm i.d., 0.25 μm film thickness) using the following oven temperature program: from 70 °C (held 2 min) to 160 °C at 40 °C/min, then to 290 °C at 8 °C/min (held 2.50 min). The injector temperature was 270 °C, the ion source temperature was 300 °C; carrier gas (helium) flow was 1.1 mL/min; the injection mode was splitless; the injection volume was 2 μL; the run time was 23.00 min, and the mass spectrometer was operated in electron ionization (EI) mode at 70 eV by full scan mode (range 40–600 *m*/*z*).

### 3.6 Synthesis of 4-Bromo-2,5-dimethoxyphenethyl Tosylate (**2**)

2-(4-Bromo-2,5-dimethoxyphenyl)­ethan-1-ol (30 mg, 0.115 mmol) was dissolved in dry dichloromethane (1 mL), and triethylamine (2.5 equiv) was added. To the stirred solution, *p*-toluenesulfonyl chloride (TsCl, 2.1 equiv) was added at room temperature, and the reaction mixture was stirred overnight under nitrogen. Reaction progress was monitored by thin-layer chromatography (TLC) using petroleum ether/ethyl acetate (7:3, v/v) as the eluent. Upon completion, the reaction mixture was diluted with dichloromethane and washed successively with 1 M HCl, saturated aqueous NaHCO_3_, and water. The organic layers were combined, dried over anhydrous Na_2_SO_4_, filtered, and concentrated under reduced pressure. The crude product was purified by flash column chromatography (petroleum ether/ethyl acetate gradient from 9:1 to 8:2) to afford the desired 4‑Bromo-2,5-dimethoxyphenethyl tosylate **2** as a white solid (32 mg, 67% yield). Compound **2** ^1^H NMR (500 MHz, CDCl_3_): δ 7.60 (2H, d, *J* = 8.3 Hz, H-2′ and H-6′), 7.24 (2H, d, *J* = 8.3 Hz, H-3′ and H-5′), 6.87 (1H, s, H-3), 6.63 (1H, s, H-6), 4.23 (2H, t, *J* = 6.5 Hz, H-8a and H-8b), 3.79 (3H, s, OCH_3_ at C-5), 3.64 (3H, s, OCH_3_ at C-2), 2.89 (2H, t, *J* = 6.5 Hz, H-7a and H-7b), 2.44 (3H, s, CH_3_ at 7′); ^1^H NMR (500 MHz, CD_3_OD): δ 7.50 (2H, d, *J* = 8.3 Hz, H-2′ and H-6′), 7.28 (2H, d, *J* = 8.3 Hz, H-3′ and H-5′), 6.94 (1H, s, H-3), 6.71 (1H, s, H-6), 4.23 (2H, t, *J* = 6.5 Hz, H-8a and H-8b), 3.75 (3H, s, OCH_3_ at C-5), 3.64 (3H, s, OCH_3_ at C-2), 2.86 (2H, t, *J* = 6.5 Hz, H-7a and H-7b), 2.44 (3H, s, CH_3_ at 7′); ^13^C NMR (125.76 MHz, CDCl_3_): δ 151.6 (C2), 149.7 (C5), 144.5 (C4′), 132.8 (C1′), 129.5 (2C, C5′ and 3′), 127.6 (2C, C2′ and 6′), 124.6 (C1), 115.5 (C3), 115.2 (C6), 109.8 (C4), 69.1 (C8), 56.8 (OCH_3_ at C5), 55.8 (OCH_3_ at C2), 30.5 (C7), 21.6 (C7′).

## 4 Conclusions

In this study, a comprehensive analytical approach combining GC–MS, LC–HRMS, and one- and two-dimensional NMR spectroscopy enabled the unambiguous identification of a previously unreported compound detected in a seized pink heart-shaped tablet. The substance was identified as 4-bromo-2,5-dimethoxyphenethyl tosylate, a putative synthetic intermediate related to the production of 2C–B derivatives.

No evidence of 2C–B itself was obtained from any of the analytical techniques employed. The seized tablet was found to contain predominantly 4-bromo-2,5-dimethoxyphenethyl tosylate, while the remaining signals detected in the NMR spectra were attributable to tablet excipients.

NMR spectroscopy allowed the clear definition of two structural fragments, while HRMS and MS/MS experiments demonstrated that all detected precursor ions corresponded to different adduct forms of a single monobrominated molecular species. Moreover, the EI GC-MS fragmentation behavior was fully consistent with the proposed structure and closely related to that of an authentic 2C–B reference standard.

The targeted synthesis of the proposed compound and the complete superimposability of the NMR and both GC- and LC-MS spectra with those of the seized material provided definitive confirmation of the structural assignment. Finally, the combined analytical and synthetic evidence converges to support the identification of the seized compound as a synthetic intermediate related to 2C–B, highlighting the relevance of integrated approaches for the characterization of emerging precursors in the context of new psychoactive substances.

Overall, these findings highlight the importance of integrated spectroscopic and spectrometric strategies for the identification of novel precursors and intermediates associated with controlled substances, which may pose significant analytical and regulatory challenges, despite lacking direct psychoactive properties.

## Supplementary Material



## Notes

^1^H and ^13^C NMR spectra of compound 2 recorded in CDCl_3_; ^1^H NMR spectrum of compound **2** recorded in CD_3_OD; 2D NMR spectra (COSY, HSQC, and HMBC) of compound **2**; ^1^H NMR spectrum of 2C–B; GC–MS spectra (EI and CI modes); LC–HRMS spectrum of compound **2** showing the [M + H]^+^ ion and MS/MS fragmentation (PDF)

## Abbreviations

NPS: new psychoactive substances
2C–B: 4-bromo-2,5-dimethoxyphenethylamine
NMR: nuclear magnetic resonance
GC-MS: gas chromatography coupled to mass spectrometry
LC-HRMS: liquid chromatography coupled to high-resolution mass spectrometry
5-HT_2A_: 2A serotonergic receptor, 5-HT_2C_, 2C serotonergic receptors
2C–B phenethyl tosylate: 4-bromo-2,5-dimethoxyphenethy tosylate
COSY: correlation spectroscopy
HSQC: heteronuclear single quantum coherence
HMBC: heteronuclear multiple bond correlation
NOESY: nuclear overhauser effect spectroscopy
1D NMR: one-dimensional nuclear magnetic resonance
2D NMR: two-dimensional nuclear magnetic resonance
