# Supplementary material for: Characterization of 4‑Bromo-2,5-dimethoxyphenethyl Tosylate, a Synthetic Intermediate of 2C–B, Identified in a Seized Pink Heart-Shaped Tablet
Source: Chem Res Toxicol. 2026 Aug 24;39(9):1780–6. doi: 10.1021/acs.chemrestox.6c00170 (PMC13599472; doi:10.1021/acs.chemrestox.6c00170)
Supplement: Supplementary file 1 [file tx6c00170_si_001.pdf]

Supporting Information

Characterization of 4-Bromo-2,5-dimethoxyphenethyl Tosylate, a Synthetic Intermediate of 2C-B, Identified in a Seized Pink Heart-Shaped Table

Sara Casati[1,2], Roberta F. Bergamaschi[1], Michele Dei Cas[3], Alessandro Ravelli[1], Lea Sicuro[4], Gabriella Roda[5], Chiara Ciccarelli[6], Luca Mollica[4], Claudio Guidotti[6], Alessio Battistini[1], Paola Rota[1,7]*

[1]Dipartimento di Scienze Biomediche, Chirurgiche ed Odontoiatriche, Università degli Studi di Milano, 20133 Milan, Italy.

[2]Fondazione IRCCS Ca' Granda Ospedale Maggiore Policlinico, 20122 Milan, Italy.

[3]Dipartimento di Scienze della Salute, Università degli Studi di Milano 20142 Milan, Italy.

4Dipartimento di Biotecnologie Mediche e Medicina Traslazionale, Università degli Studi di Milano, 20054 Segrate, Italy.

5Dipartimento di Scienze Farmaceutiche, Università degli Studi di Milano, 20133 Milan, Italy.

6Gabinetto Regionale di Polizia Scientifica per la Lombardia, Polizia di Stato, Via Fatebenefratelli 11, 20121 Milan, Italy.

7Institute for Molecular and Translational Cardiology (IMTC), 20097 San Donato Milanese, Italy.

Corresponding author e-mail: *paola.rota@unimi.it

Table of Contents

Contents Material and methods	Page Number
^{1}H NMR spectrum of the tablet containing 2C-B O-tosylate in $CDCl_3$	S3
^{1}H NMR spectrum of the tablet containing 2C-B O-tosylate in $CDCl_3$, with the excipient and residual water signals highlighted in	S4
^{1}H NMR spectrum of the tablet containing 2C-B O-tosylate in CD_3OD	S5
^{1}H NMR spectrum of the tablet containing 2C-B O-tosylate in CD_3OD, with the excipient signals highlighted in pink	S6
^{1}H NMR spectrum of the tablet containing 2C-B in CD_3OD	S7
^{1}H NMR spectrum of the tablet containing 2C-B O-tosylate in CD_3OD, overlaid with that of a tablet containing 2C-B in CD_3OD; excipient signals are highlighted in pink.	S8
^{13}C-NMR spectrum of the tablet containing 2C-B O-tosylate in $CDCl_3$	S9
COSY spectrum of the tablet containing 2C-B O-tosylate in CD_3OD	S10
HSQC spectrum of the tablet containing 2C-B O-tosylate in CD_3OD	S11
HMBC spectrum of the tablet containing 2C-B O-tosylate in CD_3OD	S12
GC–MS chromatogram and fragmentation pattern obtained using electron ionization (EI) of the tablet containing 2C-B O-tosylate	S13
LC–HRMS (TOF) chromatogram and HRMS/MS spectra of authentic 2C-B O-tosylate	S14
Table S1. Proposed fragment structure and calculated *m/z* values	S15
^{1}H NMR spectrum of authentic 2C-B O-tosylate recorded in $CDCl_3$ (high-field NMR).	S16
^{1}H NMR spectrum of the tablet containing 2C-B O-tosylate (pink trace) in $CDCl_3$, overlaid with that of authentic 2C-B O-tosylate (blue trace) in $CDCl_3$ (high-field NMR).	S17
^{13}C NMR spectrum of authentic 2C-B O-tosylate recorded in $CDCl_3$ (high-field NMR).	S18
^{13}C NMR spectrum of the tablet containing 2C-B O-tosylate (pink trace) in $CDCl_3$, overlaid with that of authentic 2C-B O-tosylate (blue trace) in $CDCl_3$ (high-field NMR).	S19
^{1}H NMR spectrum of authentic 2C-B O-tosylate recorded in $CDCl_3$ (low-field NMR).	S20
COSY spectrum of authentic 2C-B O-tosylate recorded in $CDCl_3$ (low-field NMR).	S21
NOESY spectrum of authentic 2C-B O-tosylate recorded in $CDCl_3$ (low-field NMR).	S22
GC–MS chromatogram and fragmentation pattern obtained using electron ionization (EI) of authentic 2C-B O-tosylate.	S23
Hypothetical GC-MS fragment structure	S24
Table S2. Accurate mass analysis and elemental composition assignment	S25

^{1}H NMR spectrum of the tablet containing 2C-B O-tosylate in $CDCl_3$

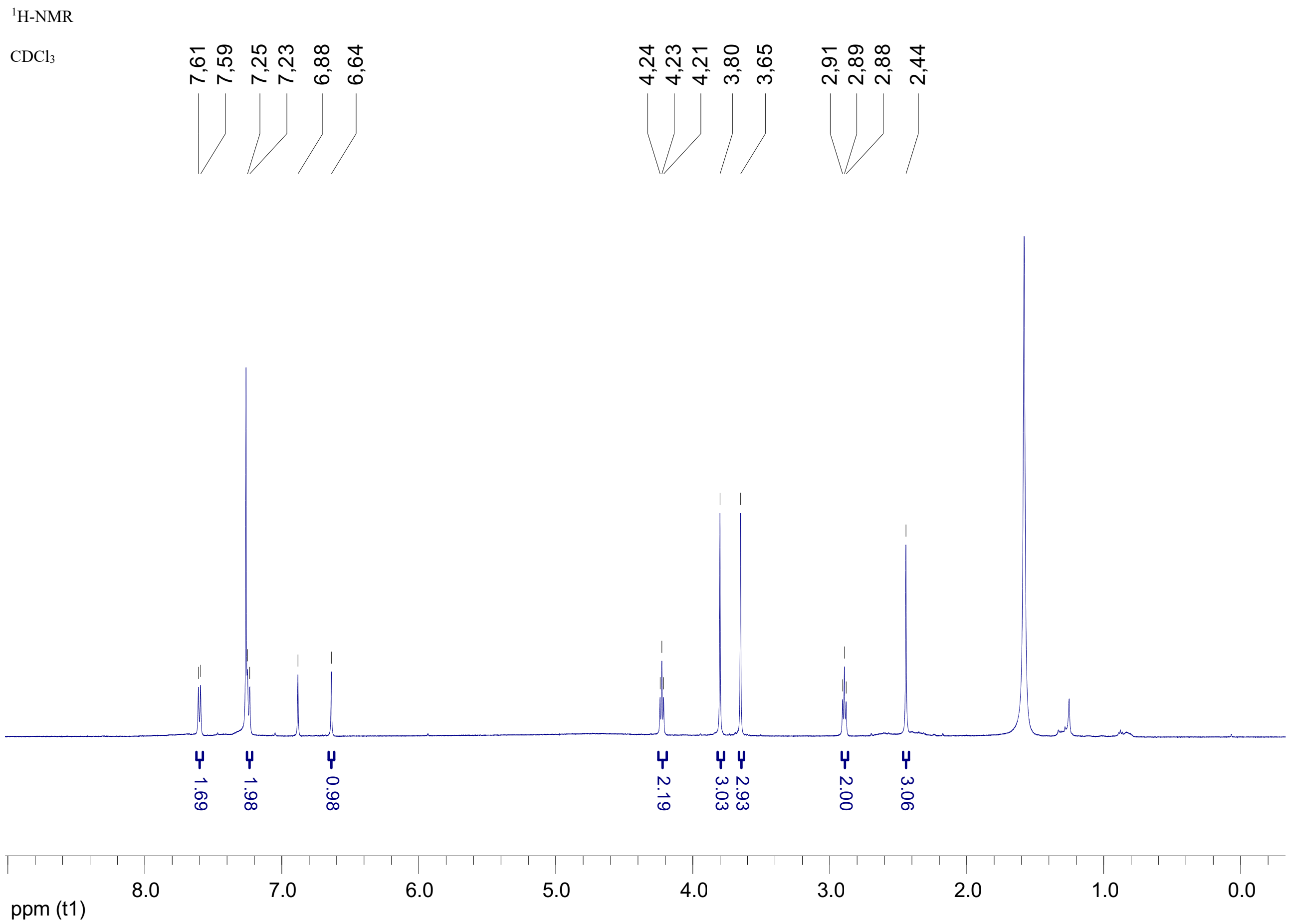

^{1}H NMR spectrum of the tablet containing 2C-B O-tosylate in $CDCl_3$, with the excipient and residual water signals highlighted in pink

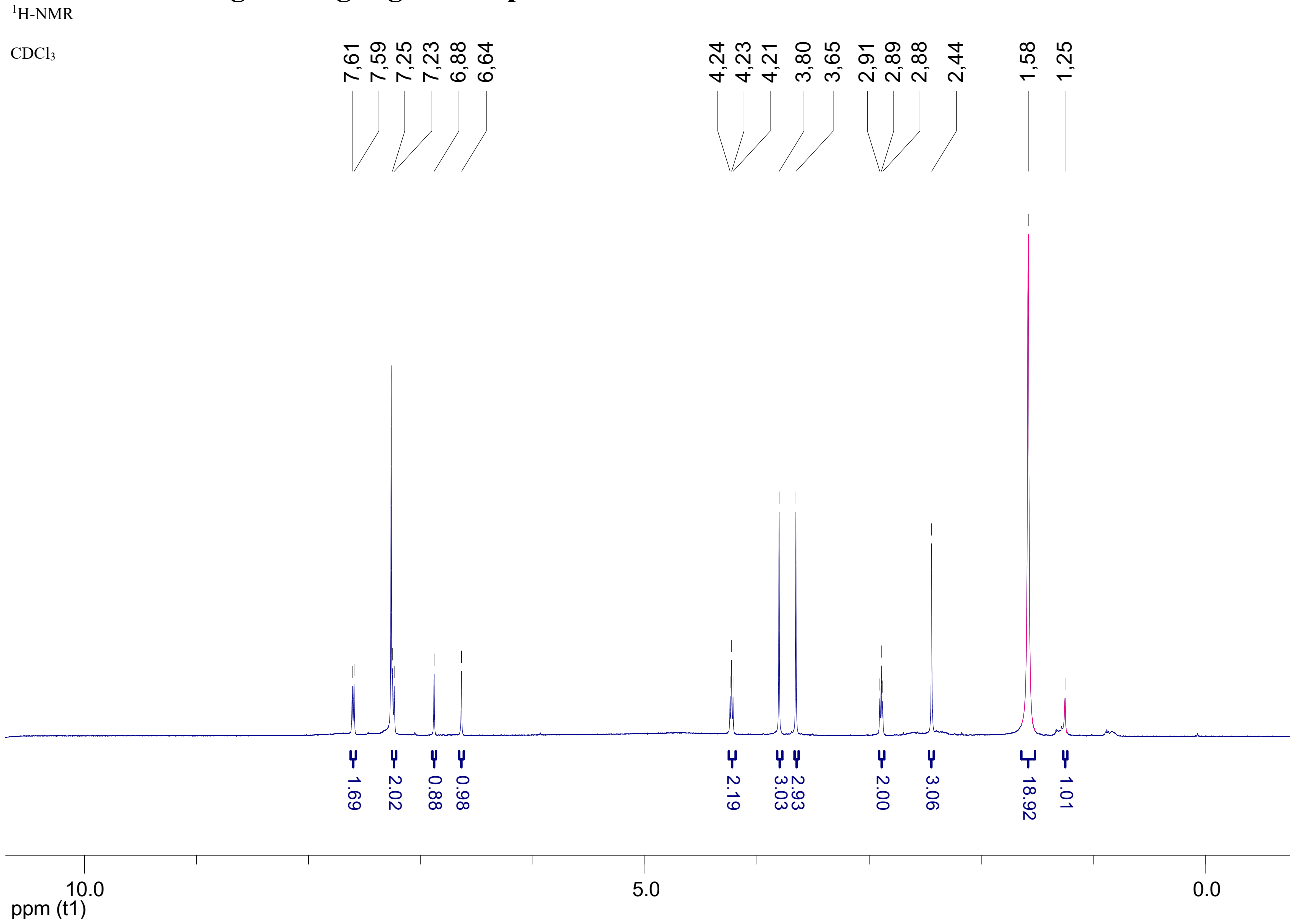

^{1}H NMR spectrum of the tablet containing 2C-B O-tosylate in CD_3OD

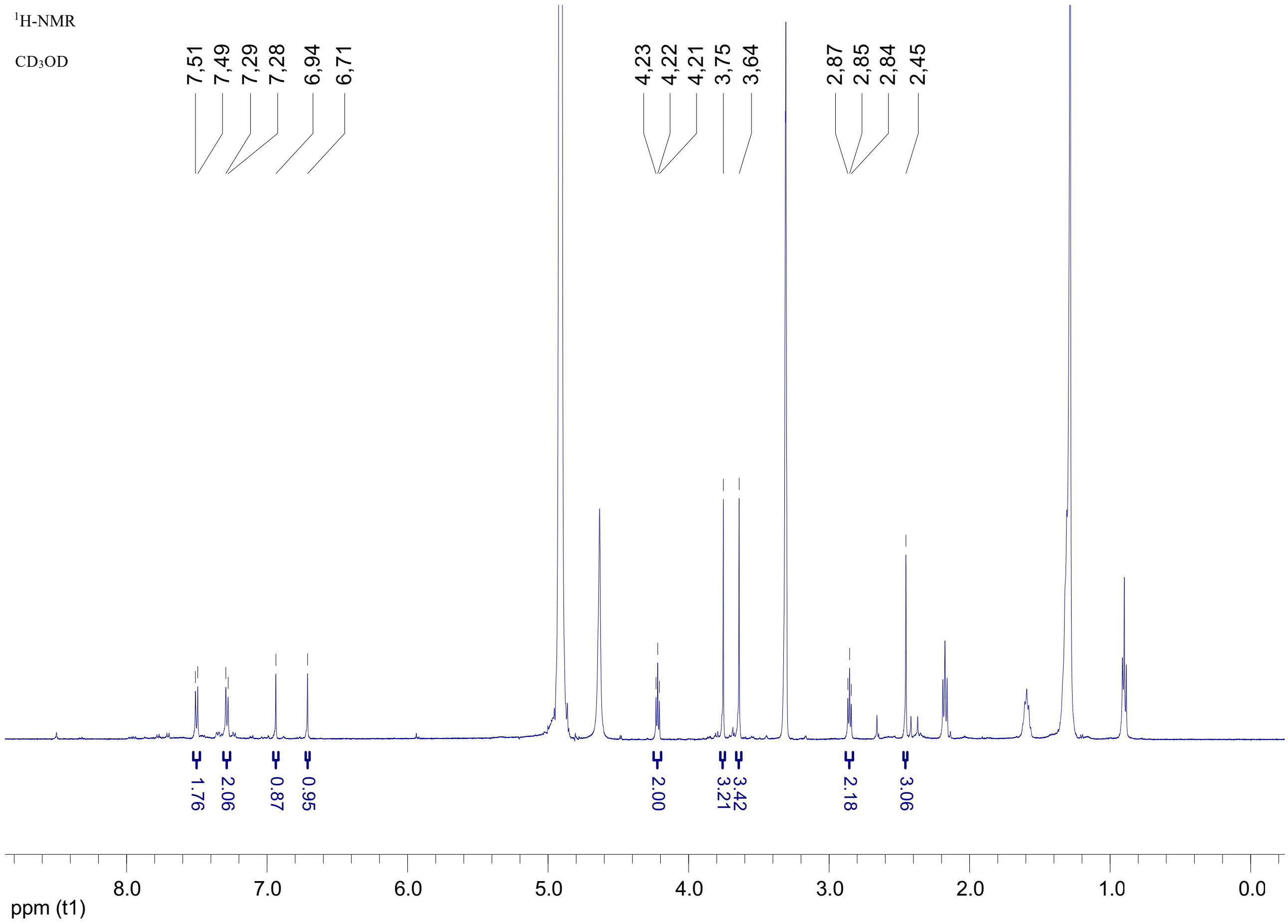

^{1}H NMR spectrum of the tablet containing 2C-B O-tosylate in CD_3OD, with the excipient signals highlighted in pink

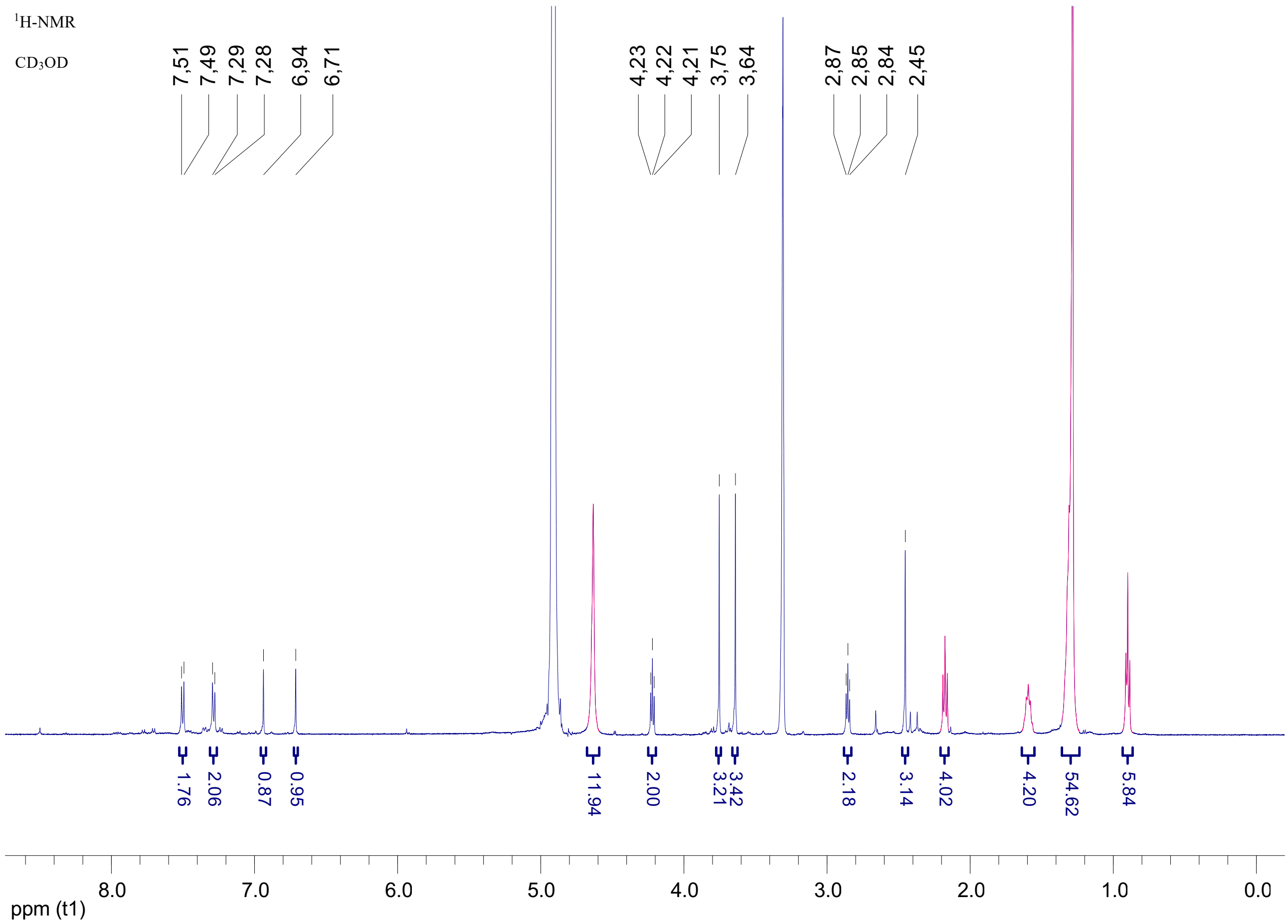

^{1}H NMR spectrum of the tablet containing 2C-B in CD_3OD

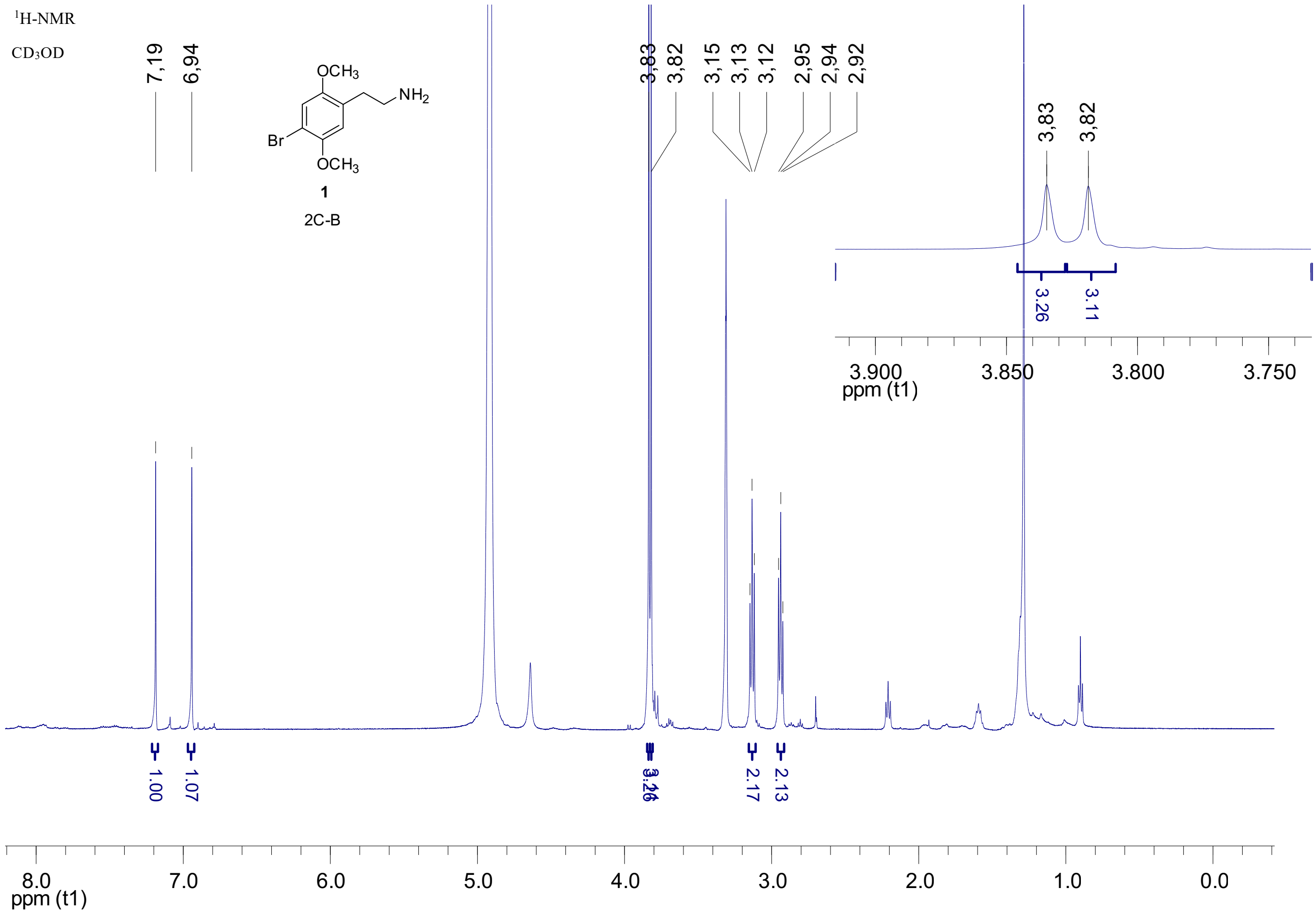

^{1}H NMR spectrum of the tablet containing 2C-B O-tosylate in CD_3OD, overlaid with that of a tablet containing 2C-B in CD_3OD; excipient signals are highlighted in pink.

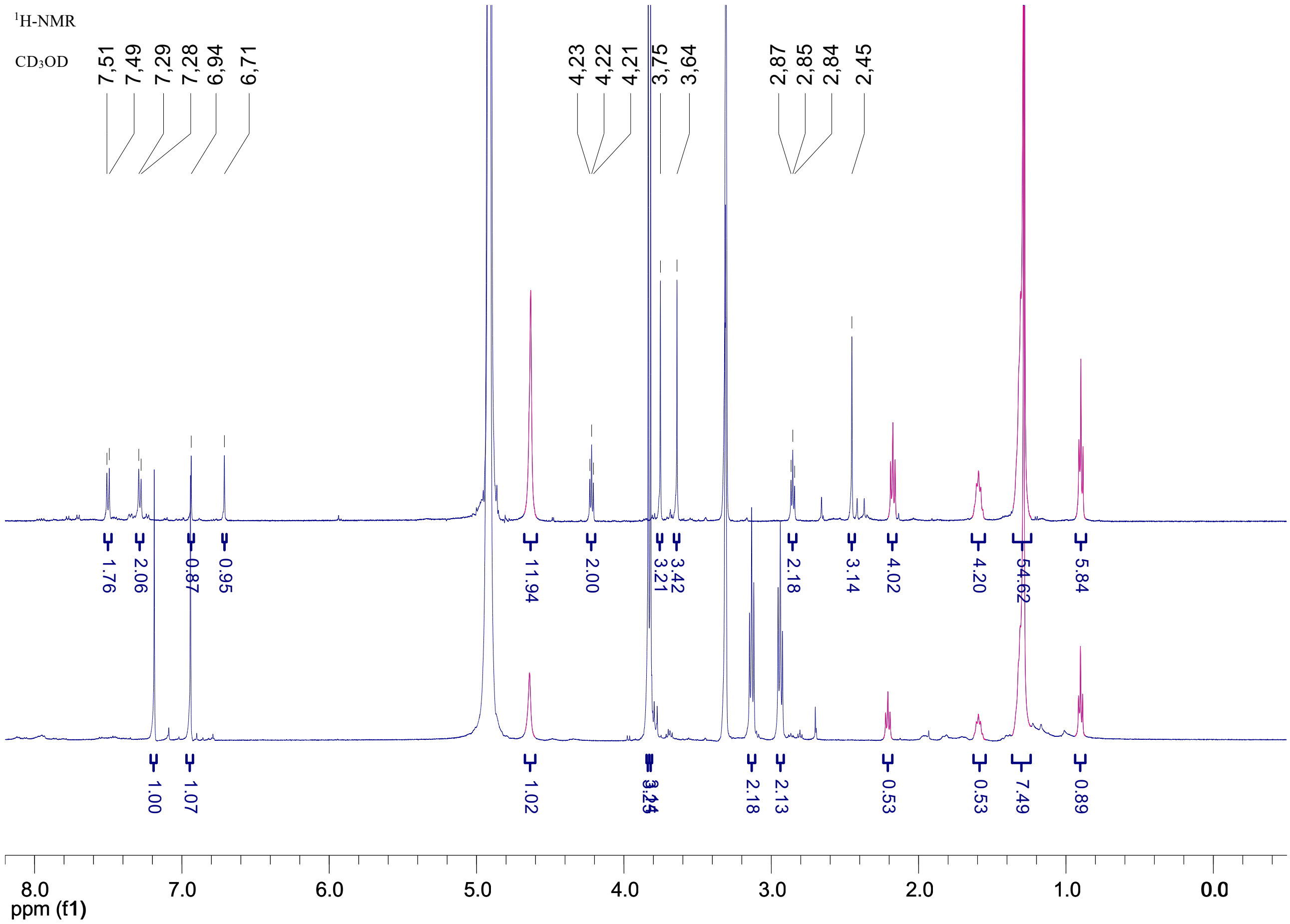

^{13}C-NMR spectrum of the tablet containing 2C-B O-tosylate in $CDCl_3$

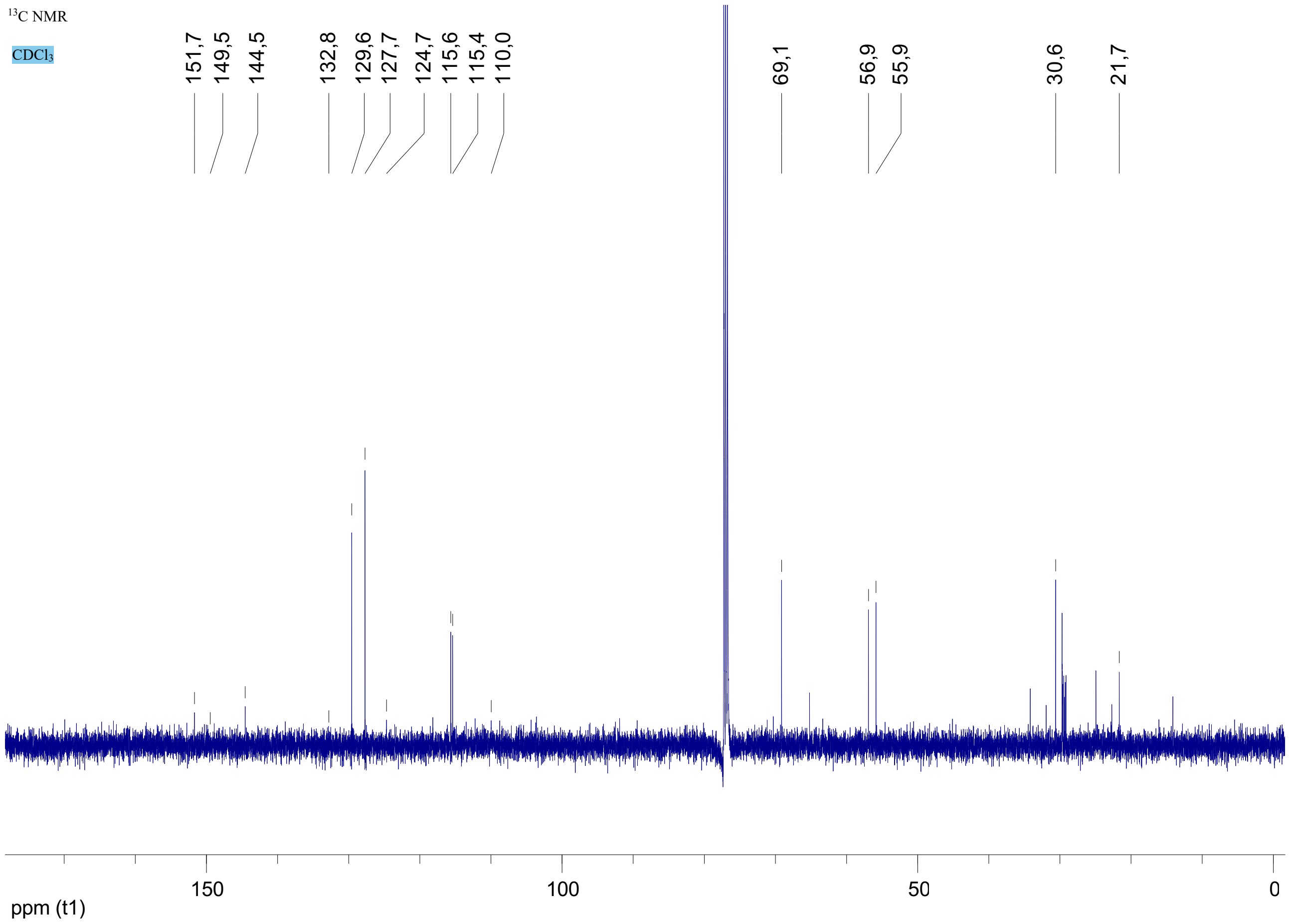

COSY spectrum of the tablet containing 2C-B O-tosylate in CD_3OD

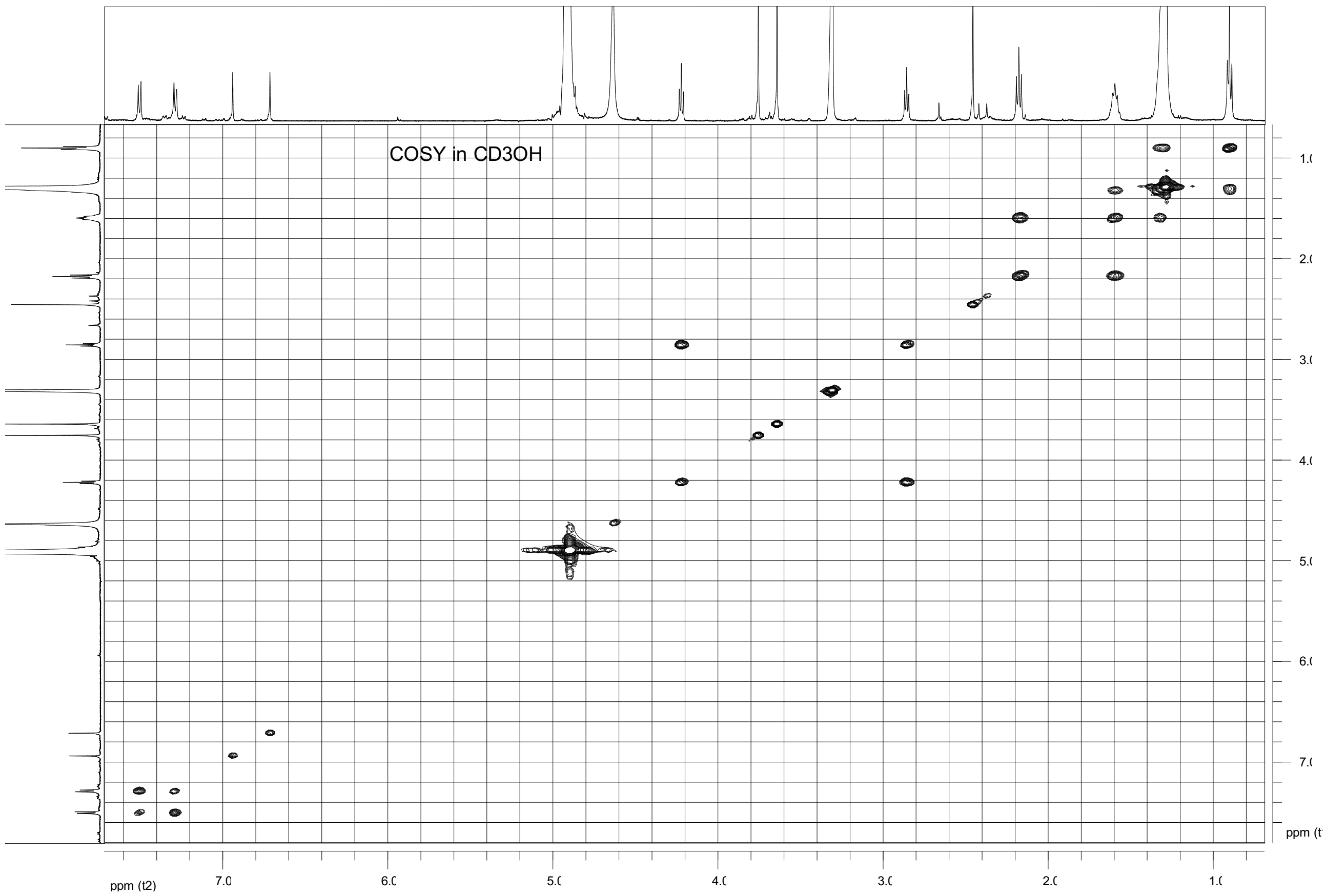

HSQC spectrum of the tablet containing 2C-B O-tosylate in CD_3OD

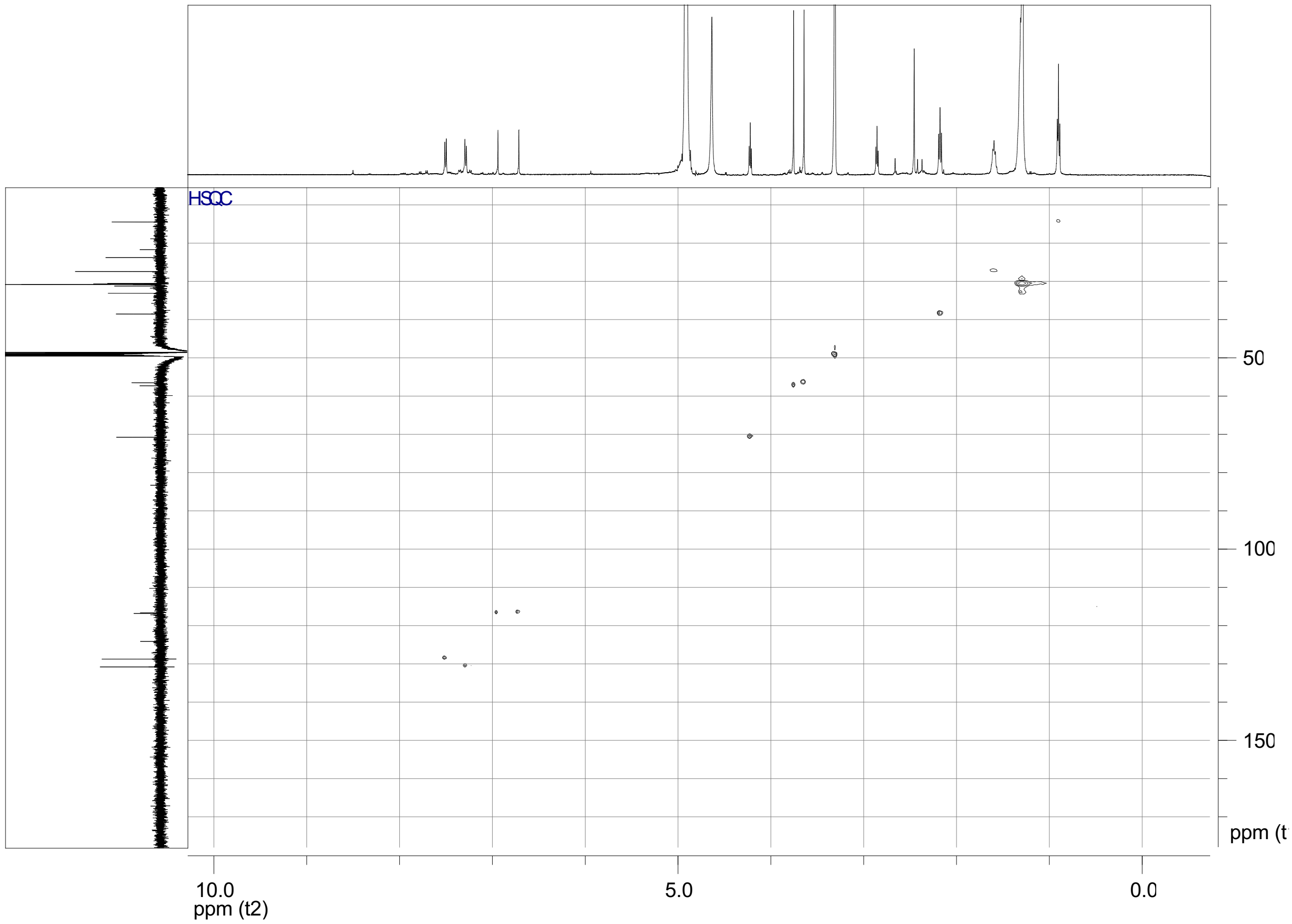

HMBC spectrum of the tablet containing 2C-B O-tosylate in CD₃OD

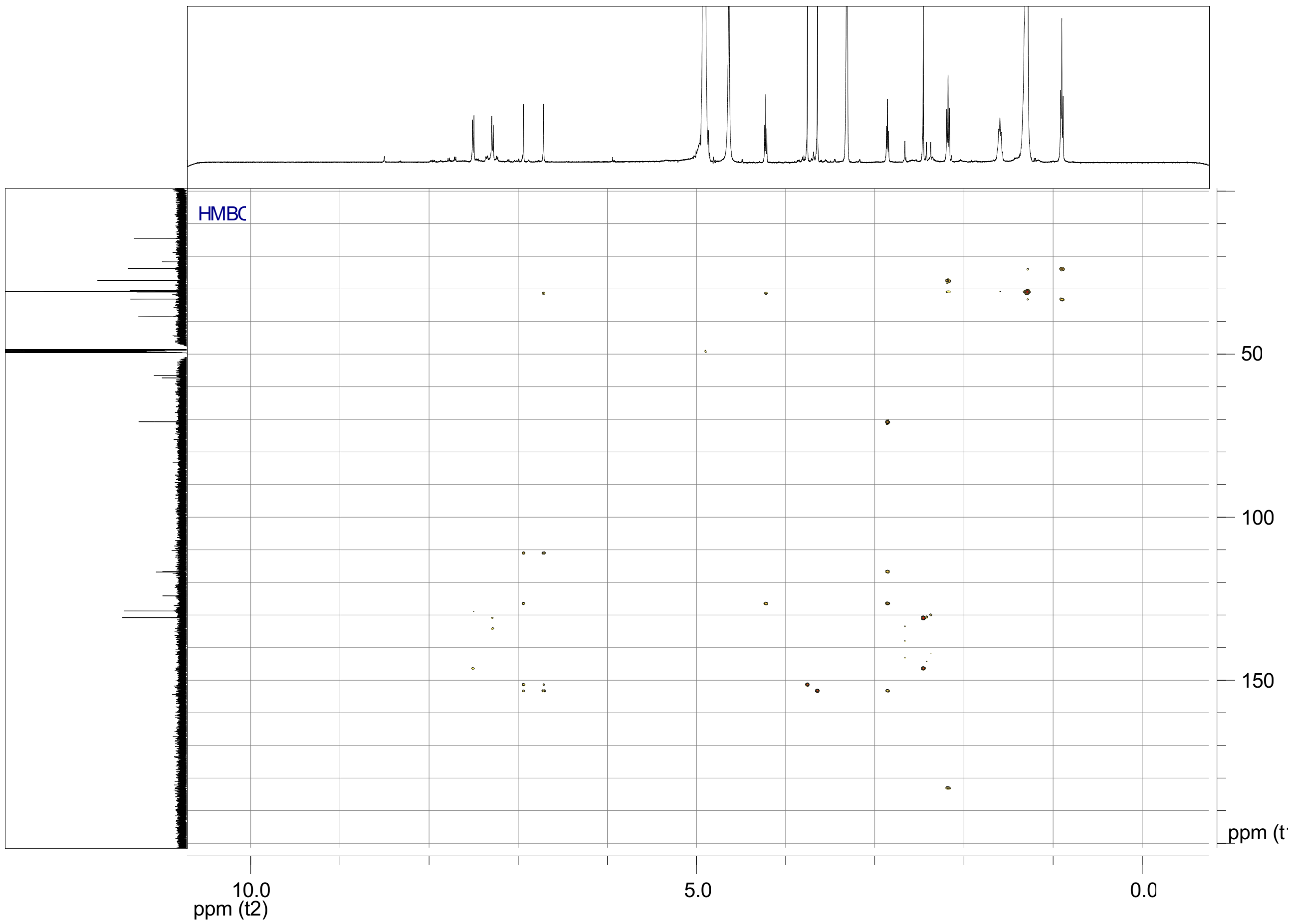

GC–MS chromatogram and fragmentation pattern obtained using electron ionization (EI) of the tablet containing 2C-B O-tosylate

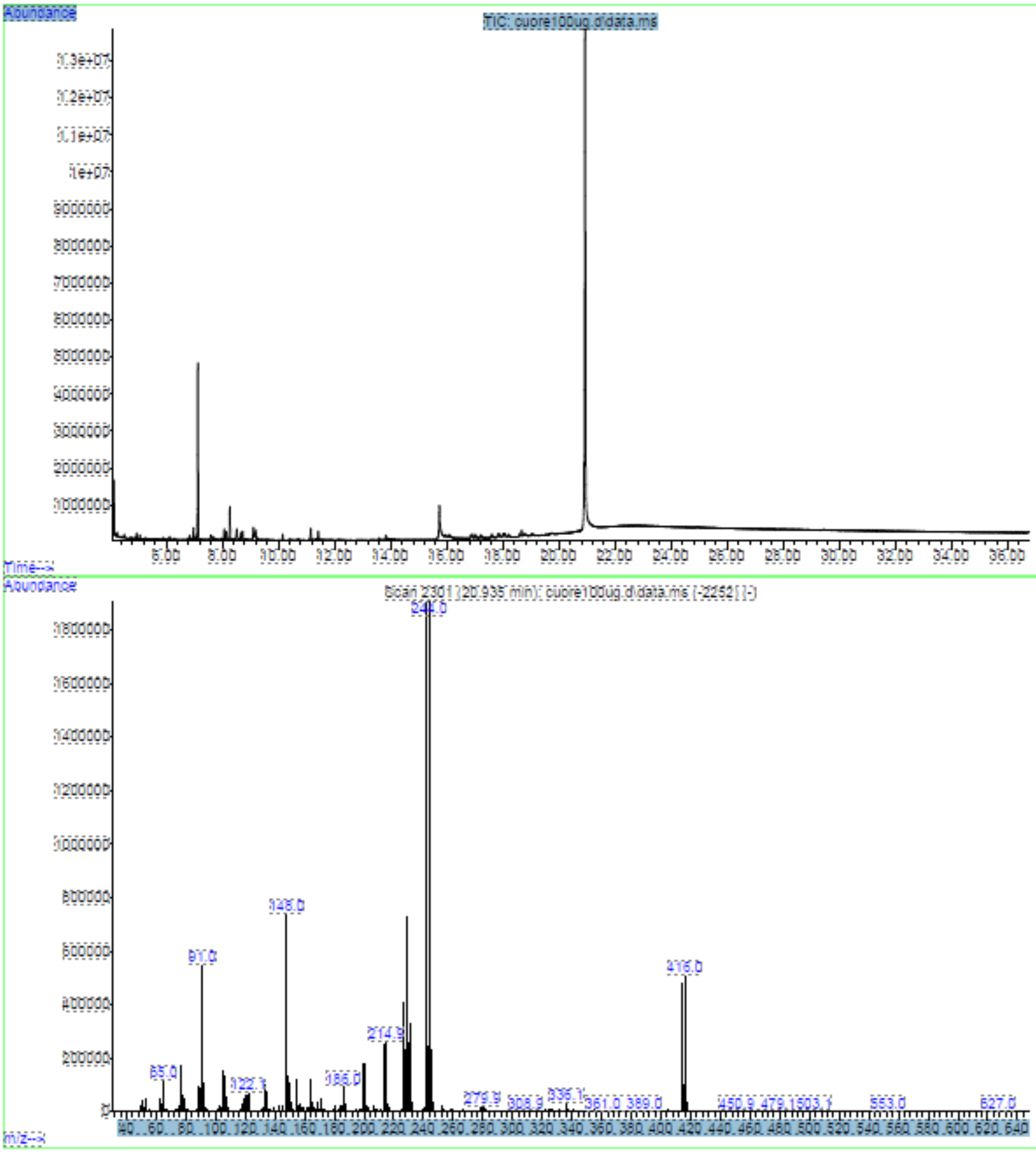

LC–HRMS (TOF) chromatogram and HRMS/MS spectra of authentic 2C-B O-tosylate

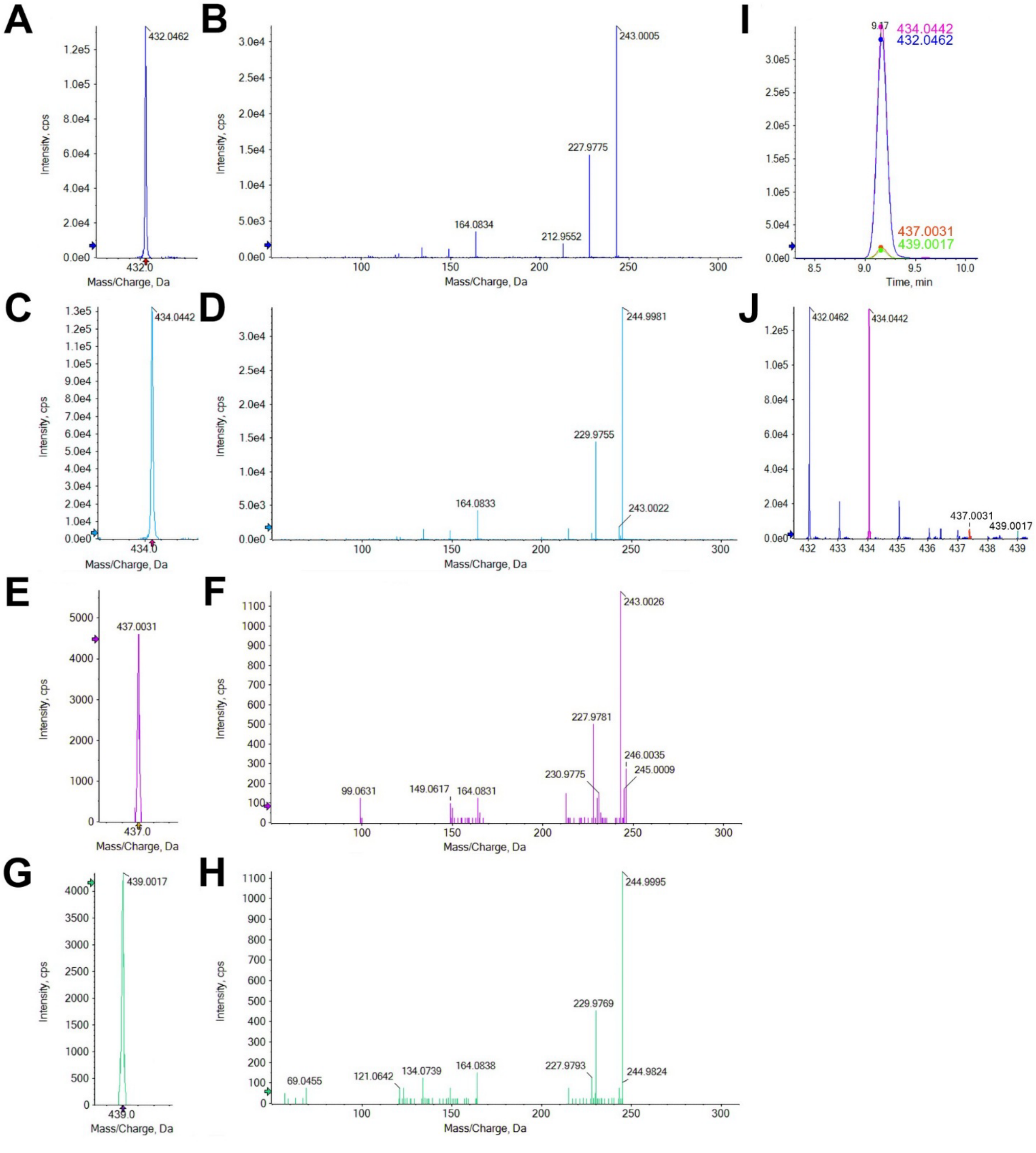

Table S1. Proposed LC-HRMS fragment structure and calculated *m/z* values

Proposed fragments/Suggested ion formula/ Calculated *m/z*
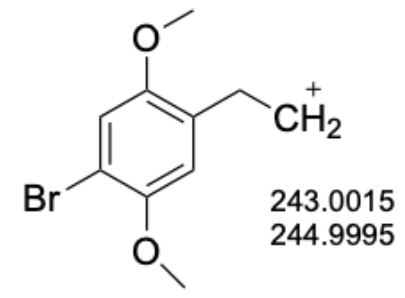 243.0015 244.9995 $C_{10}H_{12}BrO_2^+$
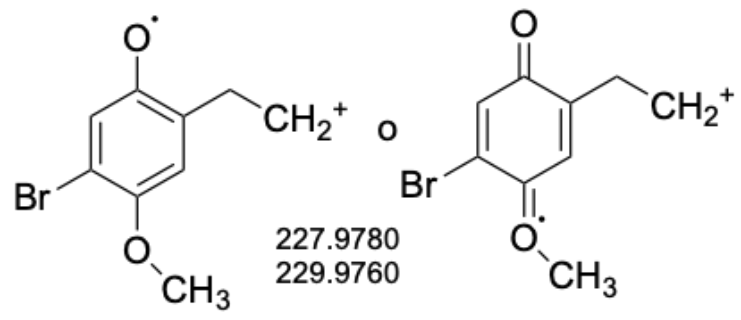 227.9780 229.9760 $C_9H_9BrO_2^{\bullet+}$ o $C_9H_9BrO_2^{\bullet+}$
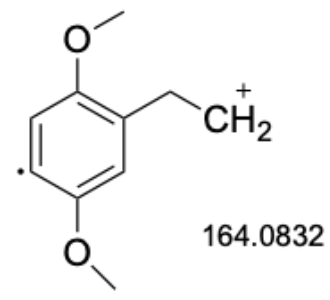 164.0832 $C_{10}H_{12}O_2^{\bullet+}$
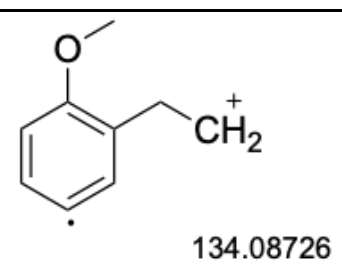 134.08726 $C_9H_{10}O^{\bullet+}$

^{1}H NMR spectrum of authentic 2C-B O-tosylate recorded in $CDCl_3$ (high-field NMR).

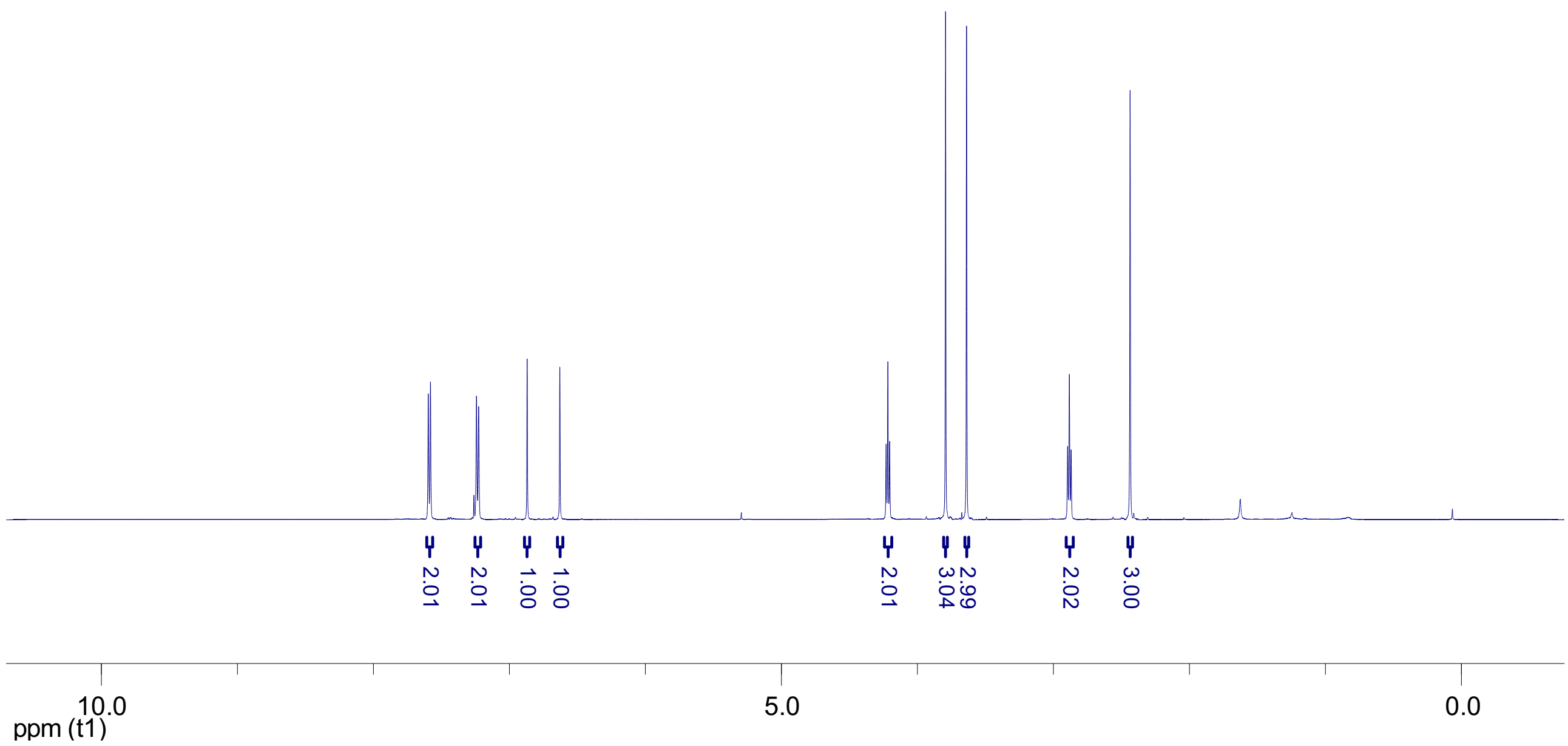

¹H NMR spectrum of the tablet containing 2C-B O-tosylate (pink trace) in $CDCl_3$, overlaid with that of authentic 2C-B O-tosylate (blue trace) in $CDCl_3$ (high-field NMR).

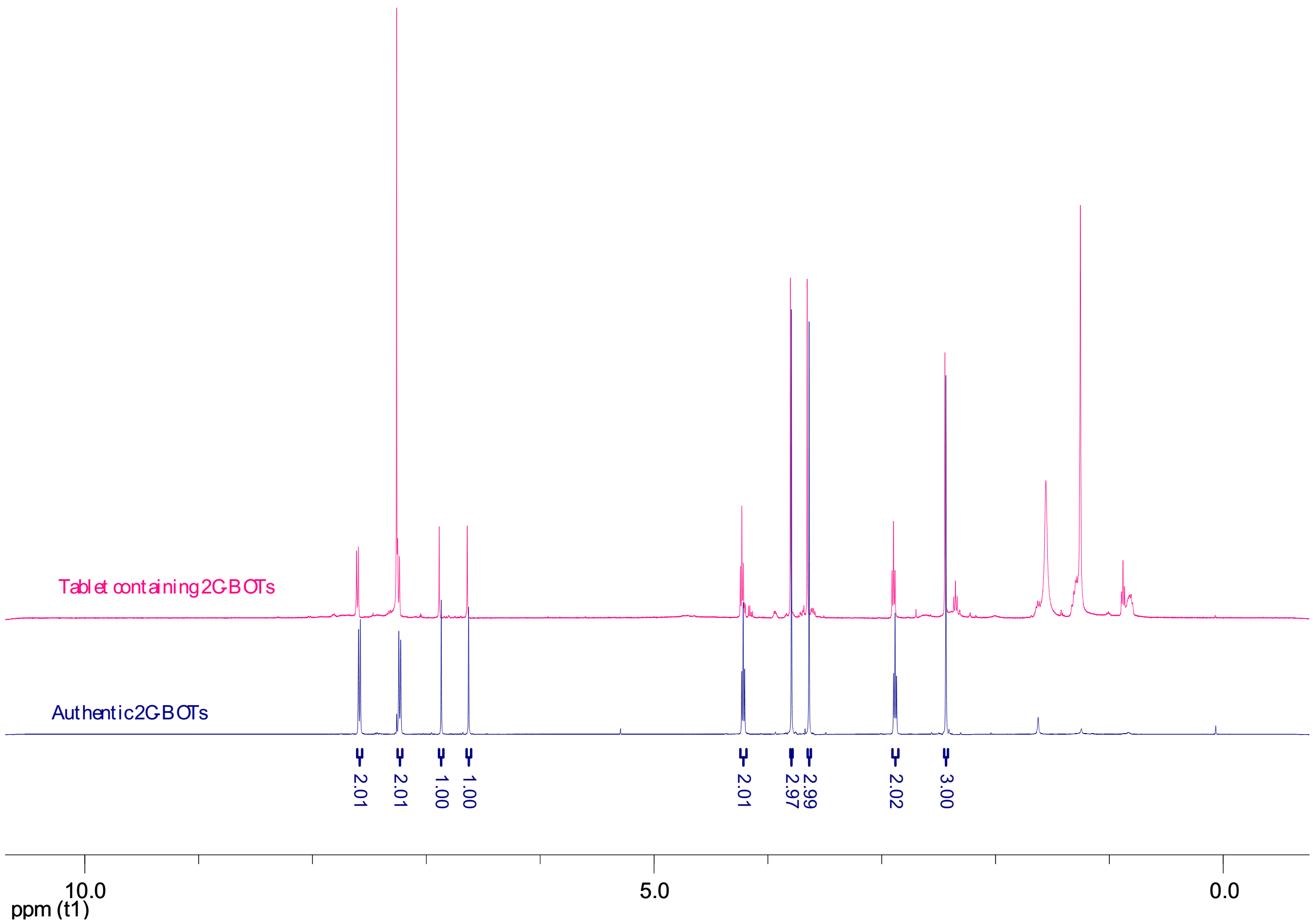

^{13}C NMR spectrum of authentic 2C-B O-tosylate recorded in $CDCl_3$ (high-field NMR).

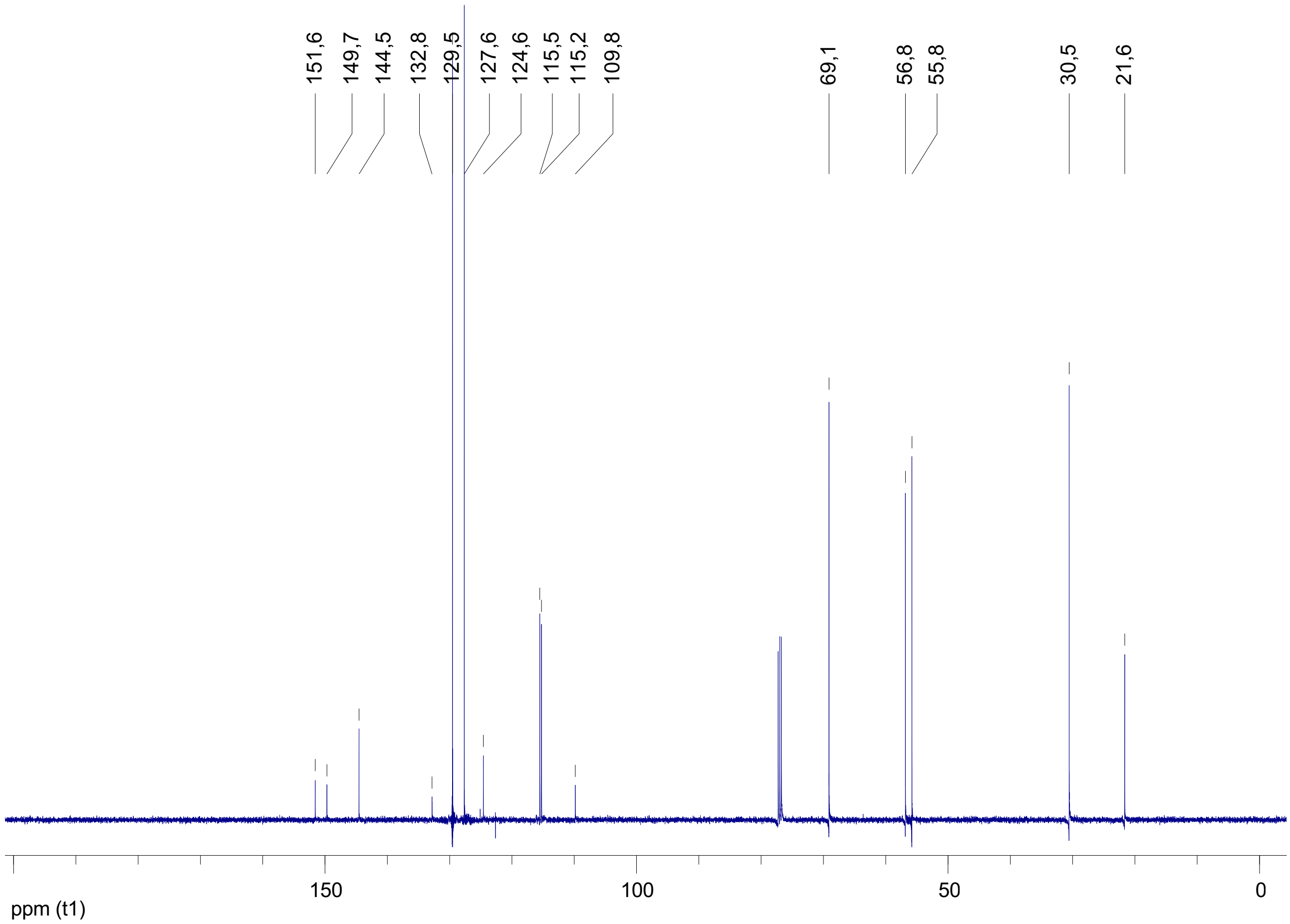

^{13}C NMR spectrum of the tablet containing 2C-B O-tosylate (pink trace) in $CDCl_3$, overlaid with that of authentic 2C-B O-tosylate (blue trace) in $CDCl_3$ (high-field NMR).

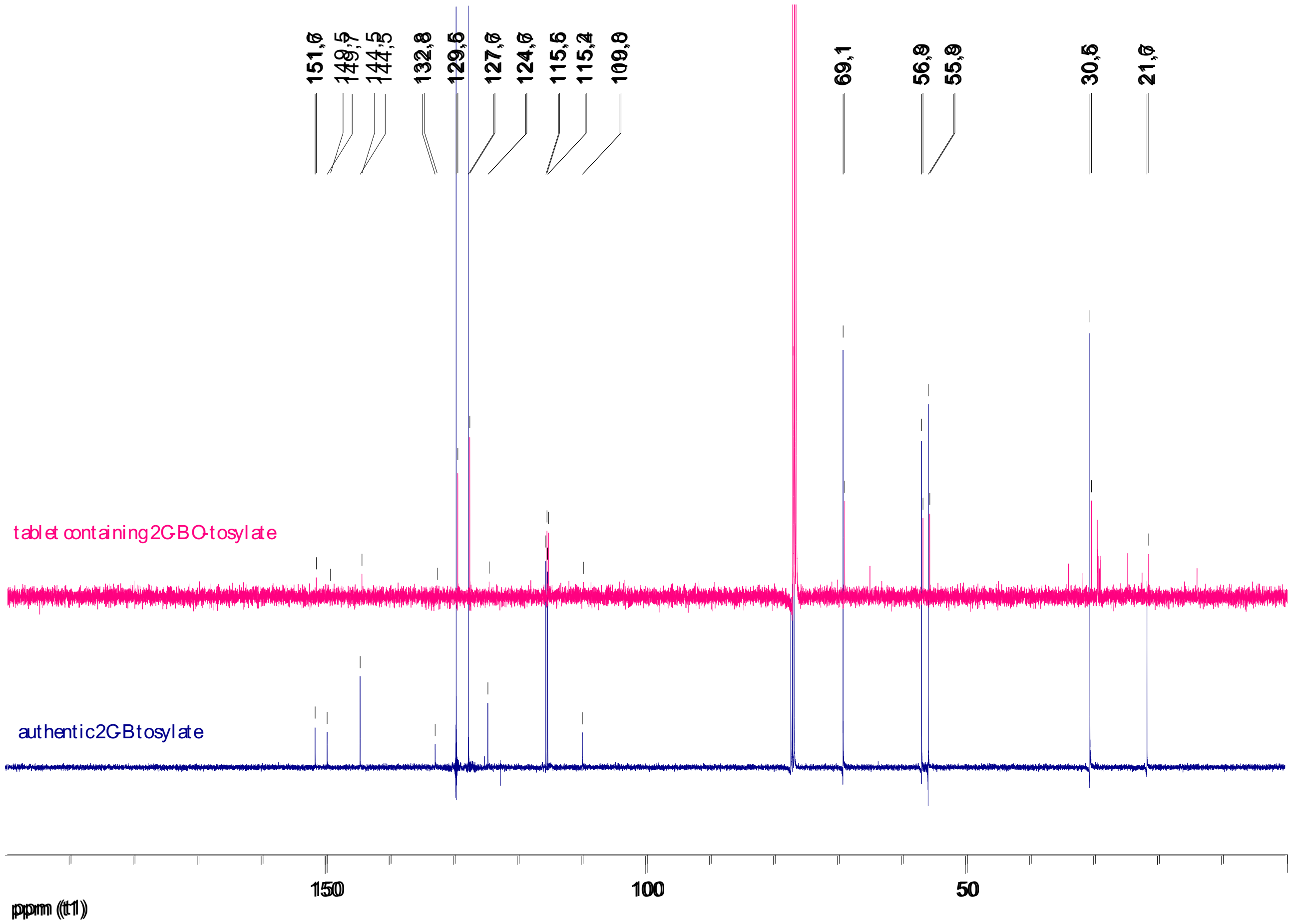

^1H NMR spectrum of authentic 2C-B O-tosylate recorded in $CDCl_3$ (low-field NMR).

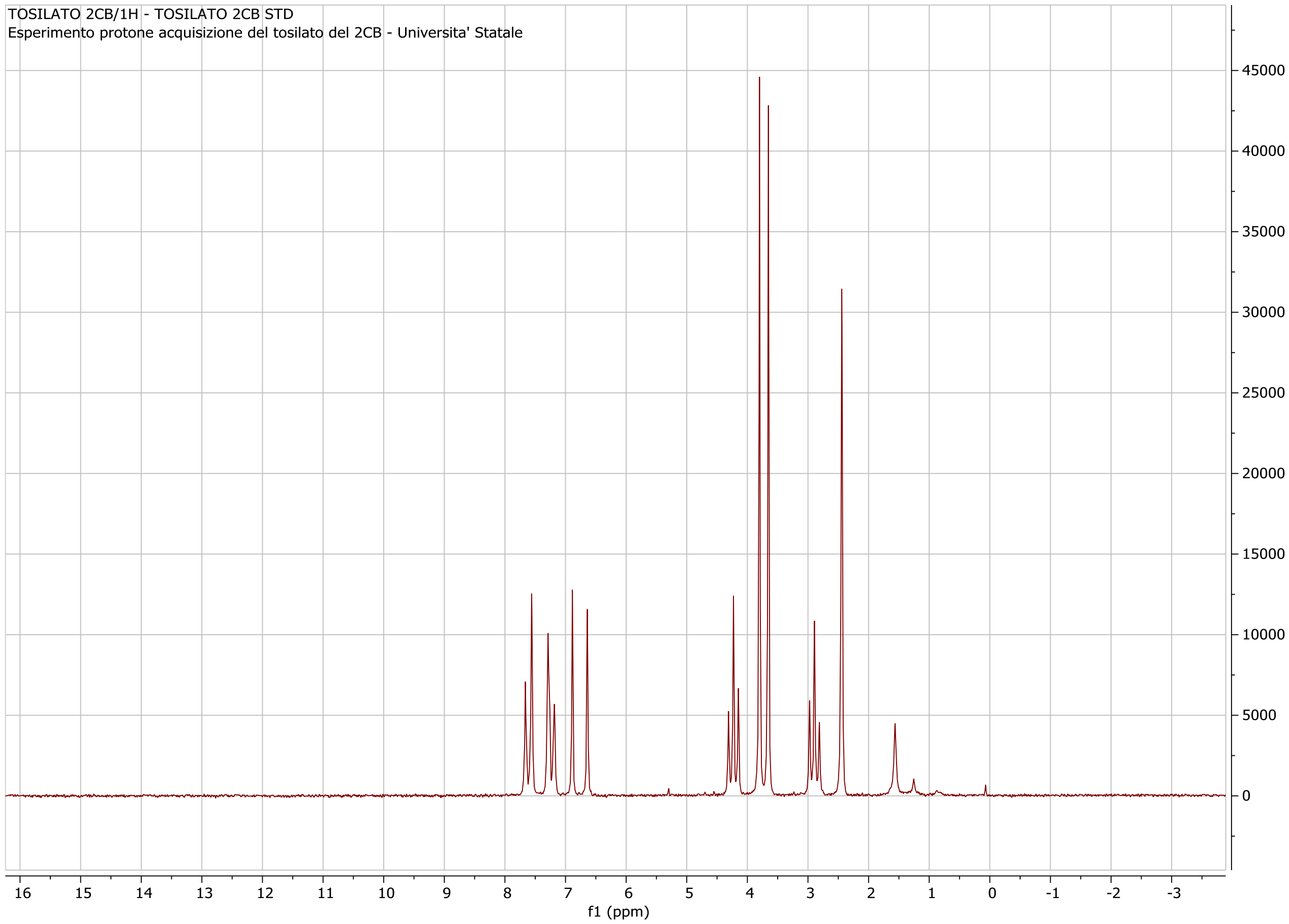

COSY spectrum of authentic 2C-B O-tosylate recorded in $CDCl_3$ (low-field NMR).

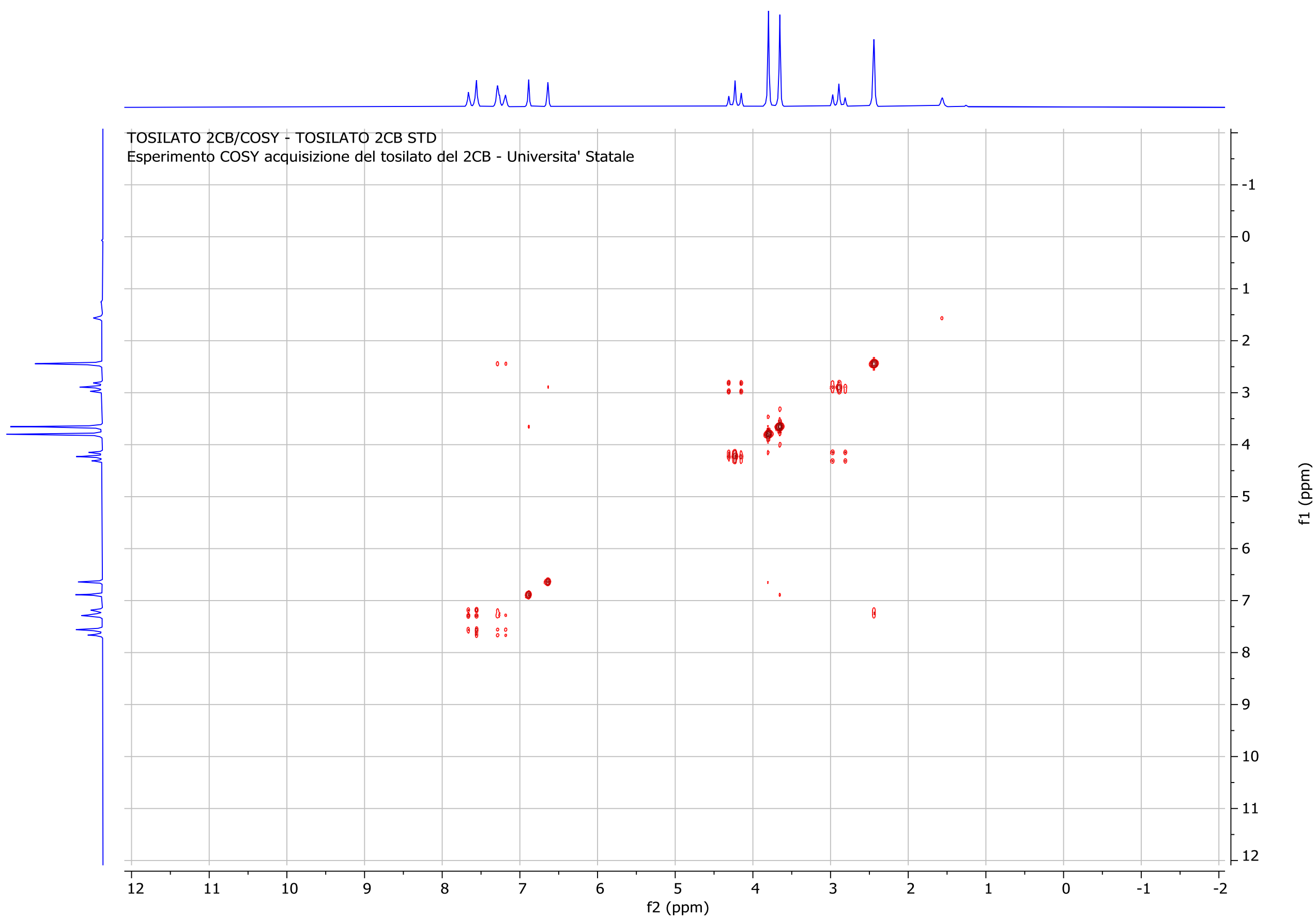

NOESY spectrum of authentic 2C-B O-tosylate recorded in $CDCl_3$ (low-field NMR).

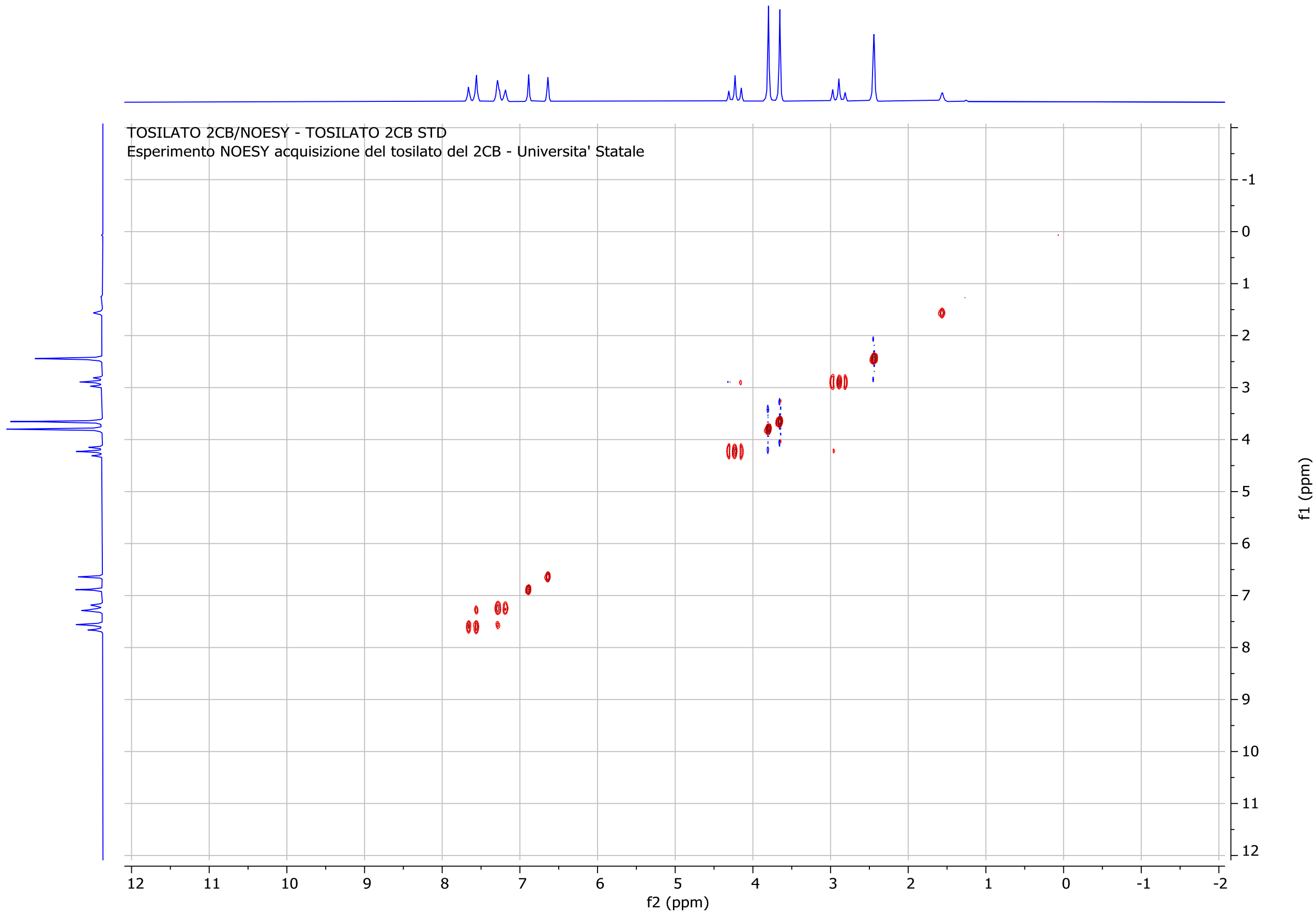

GC–MS chromatogram and fragmentation pattern obtained using electron ionization (EI) of authentic 2C-B O-tosylate

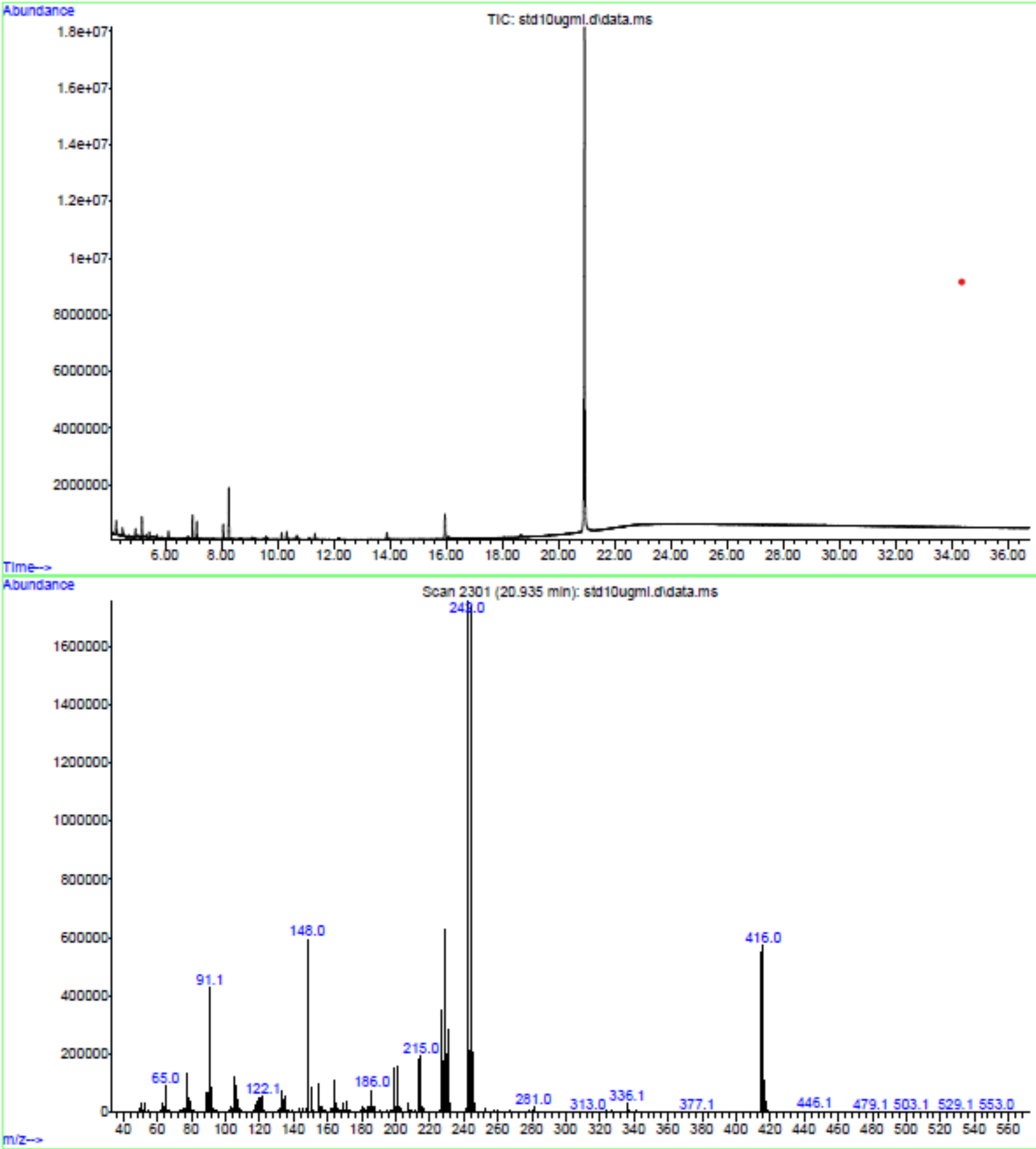

Hypothetical GC-MS fragment structure

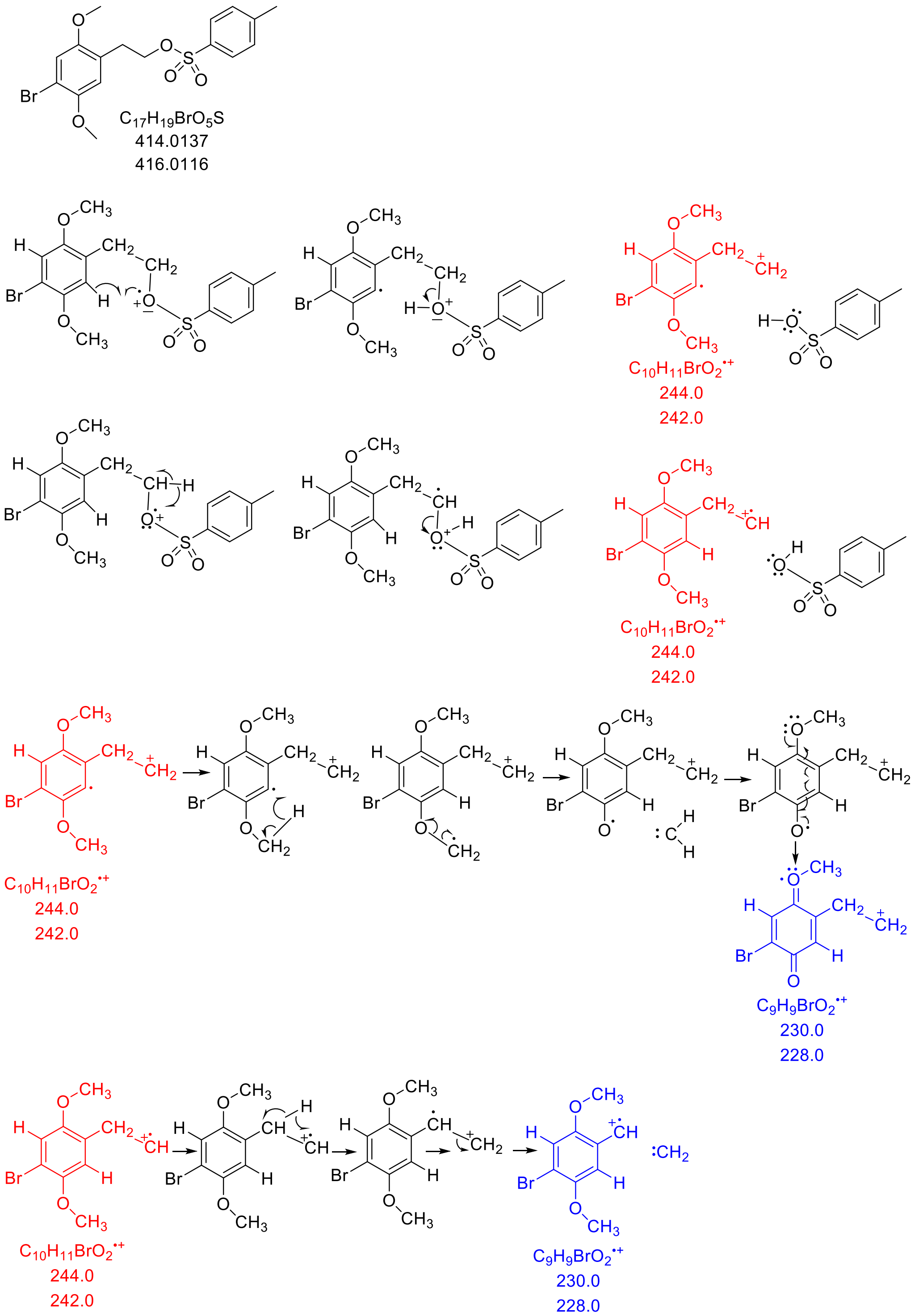

Table S2. Accurate mass analysis and elemental composition assignment

Accurate mass measurements were interpreted with a tolerance of ±0.0015 Da. Elemental compositions were evaluated considering bromine isotopic patterns (^{79}Br and ^{81}Br). Difference = experimental − calculated

**Tolerance = ±0.0015**					
Target mass = 434.0441	Minimum elemental composition: $C_{17}H_{19}O_2Br$				
Tolerance =	±0.0015				
For the calculation, the ^{81}Br isotope was used					
Formula	Calculated *m/z*	Difference	Error (ppm)		
$C_{17}H_{23}O_5SNBr^+$	434.0454	-0.0013	-3.1		
Target mass = 432.0462	Minimum elemental composition: $C_{17}H_{19}O_2Br$				
Tolerance =	±0.0015				
For the calculation, the ^{79}Br isotope was used					
Formula	Calculated m/z	Difference	Error (ppm)		
$C_{17}H_{23}O_5SNBr^+$	432.0475	-0.0013	-3.0		
Target mass = 439.0009	Minimum elemental composition: $C_{17}H_{19}O_2Br$				
Tolerance =	±0.0015				
For the calculation, the ^{81}Br isotope was used					
Formula	Calculated *m/z*	Difference	Error (ppm)		
$C_{17}H_{19}O_5SBrNa^+$	439.0008	0.0001	0.2		
Target mass = 437.0027	Minimum elemental composition: C_{17} H_{19} O_2 Br_1				
Tolerance =	±0.0015				
For the calculation, the ^{79}Br isotope was used					
Formula	Calculated m/z	Difference	Error (ppm)		
$C_{17}H_{19}O_5SBrNa^+$	437.0029	-0.0002	-0.4		
